# Macronutrient Intake during Complementary Feeding in Very Low Birth Weight Infants Comparing Early and Late Introduction of Solid Foods: A Secondary Outcome Analysis

**DOI:** 10.3390/nu16193422

**Published:** 2024-10-09

**Authors:** Melanie Gsoellpointner, Margarita Thanhaeuser, Margit Kornsteiner-Krenn, Fabian Eibensteiner, Robin Ristl, Bernd Jilma, Sophia Brandstetter, Angelika Berger, Nadja Haiden

**Affiliations:** 1 Department of Neonatology, Kepler University Hospital, Johannes Kepler University, 4020 Linz, Austria; melanie.gsoellpointner@kepleruniklinikum.at; 2 Department of Pediatrics and Adolescent Medicine, Comprehensive Center for Pediatrics, Medical University of Vienna, 1090 Vienna, Austria; margarita.thanhaeuser@meduniwien.ac.at (M.T.); margit.kornsteiner.krenn@gmail.com (M.K.-K.); fabian.eibensteiner@meduniwien.ac.at (F.E.); sophia.brandstetter@meduniwien.ac.at (S.B.); angelika.berger@meduniwien.ac.at (A.B.); 3 Center for Medical Data Science, Medical University of Vienna, 1090 Vienna, Austria; robin.ristl@meduniwien.ac.at; 4 Department of Clinical Pharmacology, Medical University of Vienna, 1090 Vienna, Austria; bernd.jilma@meduniwien.ac.at

**Keywords:** VLBW infants, solid foods, critical nutrients, macronutrient intake, complementary feeding, necrotizing enterocolitis, bronchopulmonary dysplasia, intraventricular hemorrhage

## Abstract

**Background/Objectives:** Very low birth weight (VLBW) infants may require enhanced nutrition, even during complementary feeding. However, there are limited data on macronutrient intake during this period, particularly concerning the individual timing of the introduction of solid foods in a representative VLBW infant population. **Methods:** This prospective observational study analyzed macronutrient intake in VLBW infants with a gestational age < 32 weeks based on whether solid foods were introduced early (<17 weeks corrected age (CA)) or late (≥17 weeks corrected age) Nutritional intake was analyzed using a 24 h recall at 6 weeks CA and 3-day dietary records at 12 weeks, 6, 9, and 12 months CA. **Results:** In total, 115 infants were assigned to the early and 82 to the late group. The timing of solid food introduction did not affect macronutrient intake, except for a lower fat and higher carbohydrate intake (% of energy) in the early group at 12 weeks and 6 months CA: early vs. late, fat—12 weeks: 47.0% vs. 49.0%, 6 months: 39.2% vs. 43.3%; carbohydrates—12 weeks: 44.9% vs. 43.2%, 6 months: 51.3% vs. 48.0%. Apart from docosahexaenoic acid (DHA) and arachidonic acid (AA), dietary intake recommendations were met in both groups. While nutrient intakes varied significantly between breastfed and formula-fed infants, those with comorbidities exhibited similar nutrient intake levels compared to those without. **Conclusions:** Our findings suggest adequate macronutrient intakes in VLBW infants irrespective of the timing of solid introduction. However, there is a notable need to enhance dietary intakes of DHA and AA. Future research is crucial to assess whether current nutrient intakes are sufficient for VLBW infants with comorbidities.

## 1. Introduction

The first 1000 days of life constitute a crucial period for optimizing macronutrient intake, particularly in very low birth weight (VLBW) infants [1,2]. Complementary feeding refers to the introduction of solid foods alongside breast milk or infant formula to meet the growing nutritional requirements within this critical window. While the World Health Organization (WHO) recommends that solids should be introduced at 6 months (180 days), the European Society for Paediatric Gastroenterology, Hepatology, and Nutrition (ESPGHAN) suggests introducing solids to term infants between the 17th and 26th week of life. Still, for VLBW infants, specific recommendations are lacking [3].

VLBW infants have higher nutritional needs, and insufficient macronutrient intakes during complementary feeding may impede growth and neurodevelopment [4,5]. Protein, in particular, emerges as a pivotal nutrient as an adequate intake positively influences linear growth, while an excessive intake may be linked to overweight and obesity [6,7]. Furthermore, macronutrient distribution as well as the quantity and quality of fatty acids are of major importance during development [8]. Ensuring a sufficient supply of polyunsaturated fatty acids (PUFAs) is substantial, as a deficiency can affect signal transduction and potentially result in worse neurodevelopmental outcomes [9].

Despite the importance of adequate nutritional intake during complementary feeding in former VLBW infants, the existing body of evidence is limited, with most studies conducted in regions or times no longer directly applicable [10,11]. Addressing this gap, a recent randomized, two-arm interventional trial in VLBW infants investigated differences in nutritional intake during complementary feeding between infants introduced to standardized solids early (10–12 weeks corrected age (CA) for term) versus late (16–18 weeks CA) [12,13]. While infants in the late introduction group generally displayed a lower protein intake over the first year, these differences did not attain statistical significance and fell within recommended ranges in both groups [13]. Moreover, arachidonic acid (AA) and docosahexaenoic acid (DHA) intakes did not meet current intake guidelines. This study provided valuable insights into macronutrient intake linked to the timing of solid food introduction in a standardized feeding environment. However, the exclusion of infants with neonatal comorbidities and conditions impacting stable growth from this study constrains its relevance to the wider VLBW population. Additionally, uncertainties persist as to whether current non-standardized intakes of macronutrients meet intake recommendations and whether the timepoint of introduction considering term infant guidelines (</≥17 weeks CA) affects critical nutritional intakes.

Therefore, this secondary analysis of a prospective, observational study on complementary feeding in former VLBW infants investigates macronutrient intakes up to 12 months of life with a comparison of early and late introduction before and after 17 weeks CA under non-standardized conditions. This study comprises infants with preterm birth-related comorbidities that may impact stable growth, in particular bronchopulmonary dysplasia (BPD), necrotizing enterocolitis (NEC), and intraventricular hemorrhage (IVH). We aimed to offer insights into complementary feeding in a representative VLBW infant population and nutritional intake in infants with comorbidities. Additionally, our goal was to provide nutrient intake data comparing the type of milk feeding (breastfed, formula-fed, mixed-fed).

## 2. Materials and Methods

This study is a secondary analysis of nutritional intake data collected from a prospective, observational study of VLBW infants. Infants were monitored in the outpatient clinic of the Division of Neonatology, Department of Pediatrics, Medical University of Vienna, between April 2016 and December 2021. Infants born with a birth weight < 1500 g and a gestational age < 32 weeks were included in the trial. Study exclusion criteria were Hirschsprung disease, congenital heart disease, chromosomal aberrations or major congenital birth defects. The primary objective of this study was length at 12 months CA, which was recently published by Thanhaeuser et al. [14].

The study was approved by the ethics committee of the Medical University of Vienna (EK: 1273/2016, approval date: 9 May 2016) and was registered on clinicaltrials.gov (NCT02936219, 18 October 2016). Comprehensive written informed consent was acquired from a minimum of one parent/legal guardian.

### 2.1. Group Stratification

**Figure 1:**
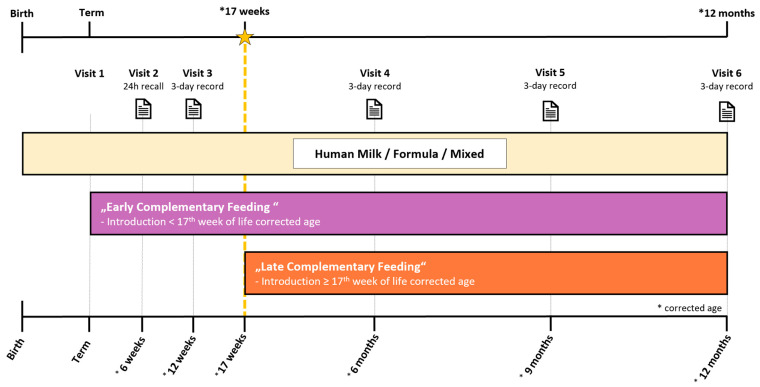
Study design.

Based on the individually chosen timepoint by parents/legal guardians for the introduction of the first complementary food, infants were classified in an early (starting solids < 17th week CA) or late group (starting solids ≥ 17th week CA) (Figure 1).

### 2.2. Study Visits, Data Collection and Evaluation

Infants with a birth weight <1500 g were regularly monitored at the neonatal outpatient clinic. A total of 6 visits were scheduled at term, 6 weeks, 12 weeks, 6, 9, and 12 months CA. Anthropometric measurements, including body weight, length, and head circumference, were obtained at the corresponding visits following a standardized operating procedure [14].

Dietary intake was calculated through a 24 h recall at 6 weeks CA and 3-day dietary records at 12 weeks, 6, 9 and 12 months CA. (Figure 1) The 3-day dietary records were recorded over three sequential days, with one of the days being on the weekend. Parents/legal guardians of the infants participating in the trial received instructions and training from a nutritionist to maintain a comprehensive food record, detailing each enteral intake at the respective timepoints. Parents/legal guardians were asked to record product brands as well as recipes of self-made meals. The dietary records were evaluated by a nutritionist employing the nut.s nutritional software (Vienna, Austria; Version II.3.1).

For breastfed infants, determining the exact amount of milk intake posed a challenge. Therefore, we relied on estimated average values for consumed breast milk, as published by Dewey et al. [15]. Detailed documentation of the infant formula utilized was included in each protocol. To ensure accurate nutrient calculation, we requested nutrient tables for all infant formulas from various manufacturers, accounting for any modifications in formulations during the study period. Nutrient ranges of infant formulas that were additionally entered into the nutritional software due to missing data or changes in formulation over time can be found in the Supplementary Materials.

The body weight noted on the relevant date of the dietary record was employed to compute protein intake per kilogram of body weight. For months lacking measurements, body weight was estimated using a linear progression based on the nearest recorded values.

### 2.3. Outcome Data

The primary outcome of this analysis was mean protein intake (g/kg/d) at 6 and 12 weeks CA as well as at 6, 9 and 12 months CA. Secondary outcomes included mean energy (kcal/d) and macronutrient distribution, i.e., fat, protein and carbohydrates as percentage of energy. Furthermore, the fatty acid profile, including mean intakes of DHA, AA, linoleic acid (LA) and α-linolenic acid (ALA), was evaluated to provide a deeper understanding of fat quality.

As an additional subgroup analysis, we investigated nutritional intakes comparing infants without preterm birth-related comorbidities and those diagnosed with BPD, NEC ≥ grade II, or IVH ≥ grade II. Moreover, this secondary outcome analysis aimed to provide nutritional intake data comparing breastfed with either formula-fed or mixed-fed infants.

Current mean nutrient intakes were contrasted with available intake recommendations for preterm infants (protein). If there were no distinct reference values available for VLBW infants, recommendations for term infants were used for comparison (macronutrient distribution, AA, DHA, LA, ALA).

### 2.4. Statistical Analysis

Nutrient intake was assessed by comparing early and late introduction of complementary feeding (</≥17th week CA). Participants who were lost to follow-up, withdrew informed consent, or did not provide sufficient dietary records were not considered for the final analysis. Dietary protocols of subjects before withdrawal of informed consent or lost to follow up were retained.

Neonatal baseline characteristics are reported as median with interquartile range (IQR). To identify differences in baseline characteristics, Pearsons chi-squared-test or the Mann–Whitney U-test were utilized.

For the statistical analysis of the primary and secondary outcome comparing early and late introduction of solids, linear mixed-effects models were used. Group (early vs. late), sex, gestational age, and nutrition at 6 weeks CA (breastmilk, formula-, or mixed-feeding) were added as fixed covariates. A random intercept was included to account for potential correlations among siblings of multiple births.

Marginal means were computed for both groups, along with their corresponding standard errors (SEs) and *p*-values to evaluate the null hypothesis of no difference between the groups.

Due to the smaller sample size, correlations between siblings of multiple birth were not considered for the subgroup analyses, and a *t*-test or the Mann–Whitney U-test were applied to detect differences in macronutrient intake between study subgroups. Statistical tests were performed comparing breastfeeding with formula feeding and breastfeeding with mixed feeding. For the comorbidity subgroup analysis, IVH, NEC and BPD were separately compared with the subgroup without comorbidities.

The figures illustrate estimated marginal means along with standard errors represented as error bars.

Additionally, the *p*-values for between-group comparisons of the same nutrient at different timepoints were adjusted using the Bonferroni–Holm method, with the adjusted *p*-values (*p*-adj) provided in the text. Adjusted *p*-values < 0.05 were deemed statistically significant. The statistical analysis was performed using R version 4.1.1 (R Core Team, 2022). The numerical results of all analyses are presented in the Supplementary Materials.

## 3. Results

### 3.1. Study Participants, Baseline Characteristics and Dietary Records

In total, 218 infants were enrolled in the study. Among them, 21 infants were categorized as drop outs due to reasons such as unavailability of information regarding the timing of solid food introduction (*n* = 4), withdrawal of parental consent (*n* = 2), screening failure (*n* = 1), missing data on the primary outcome of the study (*n* = 10) or loss to follow-up (*n* = 4). Consequently, the cohort for the initial outcome analysis comprised 197 infants, with 115 assigned to the early group and 82 to the late group [14]. While inappropriately documented or missing dietary records were further excluded, protocols of subjects before being lost to follow-up, withdrawal from informed consent or without missing data on the primary outcome were retained, resulting in 114–170 dietary protocols valid for analysis at each timepoint as displayed in Table 1.

Infants in the early group were introduced to solids at a median age of 13.7 weeks CA (IQR: 12.6–15.4), whereas infants in the late group had a median age of 19.4 weeks CA (18.3–21.7) when solids were first introduced. The median birth weight was 925 g (IQR: 718–1133) and the median gestational age was 27 + 1 weeks (IQR: 25 + 3–28 + 3) in the early group. The late group had a median birth weight of 820 g (IQR: 650–1078) and a median gestational age of 26 + 3 weeks (24 + 6–28 + 2) (Table 2).

In the late introduction group, the prevalence of infants with BPD was significantly higher (28% vs. 12%, *p* = 0.01). However, the distribution of infants with IVH and NEC was similar between the early and late groups. At 6 weeks CA, breastfeeding rates were higher in the late group (early: 24%, late: 42%, *p* = 0.01), whereas formula feeding was more prevalent in the early group (early: 57%, late: 38%, *p* = 0.01) (Table 2). Further details on the study population, obstetric and parental parameters can be found in the primary outcome report by Thanhaeuser et al. [14].

Additionally, we assessed the timepoint of introduction to solids in infants that received any kind of fortification (preterm infant formula, specially enriched formula or protein-enriched human milk) at 6 weeks CA. At 6 weeks CA, 16 infants (18.6%) in the early group received either preterm infant formula, specially enriched formula, or protein-enriched human milk, compared to 9 infants (15%) in the late group. Among the infants who received fortification, the median age at which solids were introduced was 16.3 weeks CA (IQR: 13.3–19.0). For infants who did not receive enriched formula or breastmilk, the median age for the introduction of solids was 16.1 weeks CA (IQR: 13.6–19.0); the difference was not statistically significant (*p* = 0.94).

### 3.2. Macronutrient Intake Comparing Early Versus Late Introduction of Solid Foods

#### 3.2.1. Dietary Protein Intake

**Figure 2:**
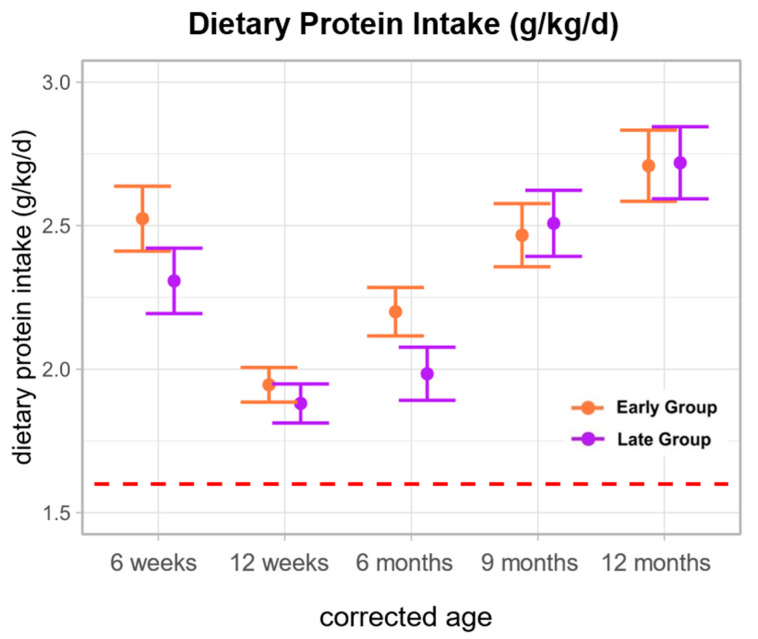
Protein Intake (g/kg/d). The red line indicates the reference value for protein intake in preterm infants (1.6 g/kg/d). Data are presented as the estimated marginal mean and standard error.

Dietary protein intake (g/kg/d) was similar among the early and late group throughout the observation period [6 weeks–12 months; early—2.52, 1.95, 2.20, 2.47, 2.71 g/kg/d; late—2.31, 1.88, 1.98, 2.51, 2.72 g/kg/d]. Notably, mean dietary protein intake experienced a decline at 12 weeks CA in both groups but demonstrated an upward trend at 6 months CA, which was more pronounced in the early group [early: 2.20 g/kg/d (SE ± 0.08), late: 1.98 g/kg/d (SE ± 0.09)]. Throughout the study period, the recommended protein intake of 1.6 g/kg/d was consistently met [16] (Figure 2).

#### 3.2.2. Dietary Energy Intake and Macronutrient Distribution

**Figure 3:**
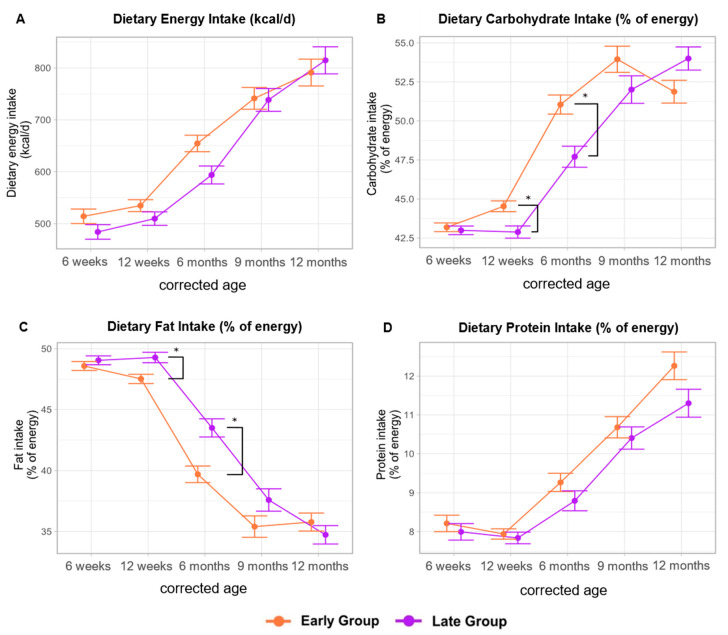
Dietary energy intake (kcal/d) and macronutrient distribution (% of energy). Significant differences (adjusted *p*-value < 0.05) are marked with *. Data are presented as the estimated marginal mean and standard error.

After adjusting for multiple testing, we did not observe a significant difference in mean energy intake between the early and the late group throughout the observation period (Figure 3A). Nevertheless, the early group tended to have a higher mean energy intake until 9 months CA with levels ranging from 514 (SE ± 14) kcal/d at 6 weeks CA up to 791 (SE ± 16) kcal/d at 12 months CA. The late group had mean energy intakes between 484 (SE ± 14) kcal/d and 815 (SE ± 26) kcal/d at 6 weeks and 12 months CA, respectively. (A) Except for 6 weeks CA, the mean energy intake in the late group was deemed adequate at all examined timepoints (reference values for term infants—0–3 months: 500–550 kcal/d; 4–12 months: 600–700 kcal/d) [17].

While the percentage of energy derived from protein remained consistent across the groups, there were notable variations in the proportional intake of carbohydrates and fat throughout the study period. Specifically, the early group exhibited a significantly higher mean proportional carbohydrate intake at 12 weeks [early: 44.5% (SE ± 0.35), late: 42.9% (SE ± 0.39); *p*-adj = 0.003] and 6 months CA [early: 51.1% (SE ± 0.61), late: 47.7% (SE ± 0.67); *p*-adj = 0.002] (Figure 3B). Conversely, infants in the late group demonstrated a contrasting trend in fat intake, with significantly higher mean proportional fat intake at 12 weeks [early: 47.5% (SE ± 0.36), late: 49.3% (SE ± 0.43); *p*-adj = 0.005] and 6 months CA [early: 39.7% (SE ± 0.68), late: 43.5% (SE ± 0.75); *p*-adj = 0.001] (Figure 3C). The proportional mean protein intake ranged from 8.2% at 6 weeks to 12.3% at 12 months CA in the early group and 8.0% to 11.3% in the late group, respectively (Figure 3D).

#### 3.2.3. Polyunsaturated Fatty Acid Profile

No differences were found in any of the variables pertaining to the fatty acid profile (DHA, AA, LA, ALA, LA/ALA) between the groups throughout the observation period.

**Figure 4:**
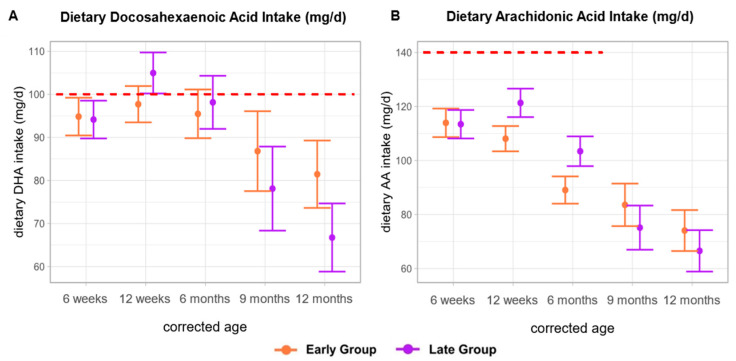
Dietary docosahexaenoic acid intake (DHA) and arachidonic acid intake (AA) (mg/d). The red lines indicate the reference values for DHA (0–12 months: 100 mg) and AA intake (0–6 months: 140 mg/d); no AA recommendations are available for 6–12 months. Data are presented as the estimated marginal mean and standard error.

According to current guidelines, a DHA intake of 100 mg/d is recommended throughout the first year of life [18]. In the early group, this recommendation was not met at any of the investigated timepoints with the lowest intake at 12 months CA [81.4 mg/d (SE ± 7.8)]. In the late group, recommendations were fulfilled only at 6 weeks CA [105.0 mg/d (SE ± 4.8)]. Moreover, the mean DHA intake declined as solid foods constituted a higher percentage of the daily diet (Figure 4A).

We found that the mean dietary AA intake fell below the recommended daily AA intake (0–6 months: 140 mg/d [19]) at all of the investigated timepoints. The highest intake levels were observed at 6 weeks CA in the early group [114.0 mg/d (SE ± 5.3)] and at 12 weeks CA in the late group [121.0 mg/d (SE ± 5.3)]. Notably, the mean AA intakes demonstrated a consistent decrease as solid foods increased in the infants’ diets (Figure 4B).

The mean dietary LA intakes consistently surpassed the recommended daily intake levels (0–3 months: 4.0% of energy; 4–12 months: 3.5% of energy) across the entire observation period [17]. Notably, the mean LA intake (% of energy) was highest at 12 weeks CA in both groups [early: 6.2% (SE ± 0.10), late: 6.0% (SE ± 0.11)]. The mean dietary LA intake decreased as infants were gradually weaned from breast milk or infant formula, reaching levels of 5.0% (SE ± 0.17), and 4.7% (SE ± 0.17) at 12 months CA in the early and late group, respectively.

The proportional mean intake of ALA (% of energy) exceeded the dietary intake recommendations (0–12 months: 0.5% of energy) at all measured timepoints in both groups [17]. The mean ALA intake remained constant throughout the observation period, irrespective of the proportional quantity of breast milk, infant formula, and solids. The mean ALA intakes were 0.71% (SE ± 0.02) and 0.66% (SE ± 0.03) at 6 weeks and 0.77% (SE ± 0.04) and 0.67% (SE ± 0.04) at 12 months CA in the early and late group, respectively.

The late group experienced a slightly higher LA/ALA ratio until 6 months CA, however, without statistical significance [6 weeks CA—early: 9.1 (SE ± 0.2), late: 9.6 (SE ± 0.2); 12 weeks CA—early: 8.7 (SE ± 0.2), late: 9.2 (SE ± 0.2); 6 months CA—early: 7.0 (SE ± 0.3), late: 7.7 (SE ± 0.3)]. Except for 6 weeks and 12 weeks CA in the late group, LA/ALA levels were close to the recommended values (0–3 months: 8:1; 4–12 months: 7:1) in both groups [17].

### 3.3. Macronutrient Intake in Infants with Preterm Birth-Related Comorbidities

We conducted subgroup analyses to detect differences in nutrient intake between infants without preterm birth-related comorbidities and those with BPD, NEC ≥ grade II, or IVH ≥ grade II.

**Figure 5:**
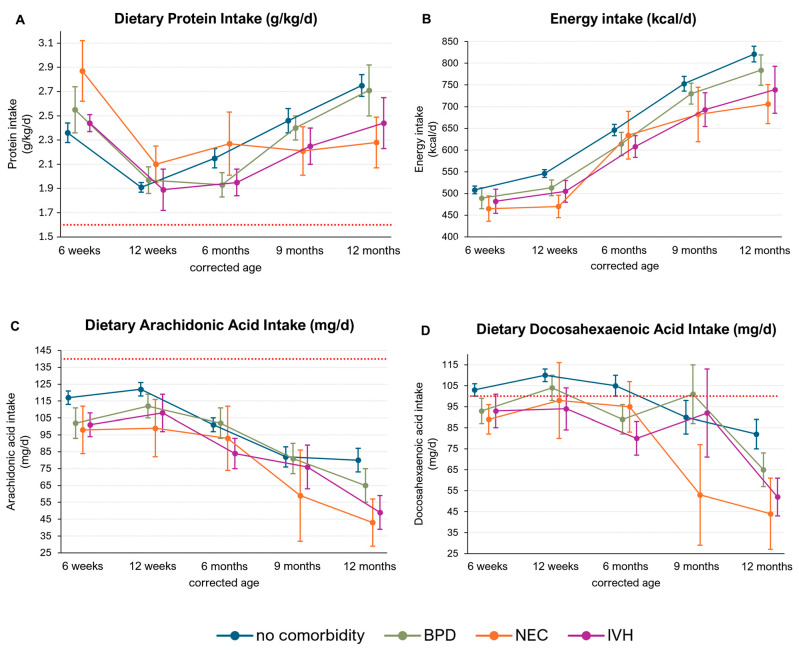
Protein (g/kg/d), energy (kcal/d), docosahexaenoic acid (DHA) and arachidonic acid (AA) (mg/d) intake in infants with comorbidities. The red lines represent intake recommendations for the respective nutrient. Data are presented as the mean and standard error.

The mean protein intake (g/kg/d) was similar among the subgroups, ranging from 1.93 to 2.71 g/kg/d in infants with BPD, 2.10 to 2.87 g/kg/d in infants with NEC, and 1.89 to 2.44 g/kg/d in those with IVH (Figure 5A).

We observed a lower energy intake in infants with IVH, NEC, and BPD compared to those without comorbidities. However, these differences did not reach statistical significance (no comorbidity: 508–821 kcal/d; BPD: 489–784 kcal/d; NEC: 465–706 kcal/d; IVH: 482–739 kcal/d) (Figure 5B).

No significant differences were detected in AA intake between the subgroups. Nonetheless, there was a trend towards decreased AA intake in infants with NEC and IVH (no comorbidity: 80–117 mg/d; NEC: 43–98 mg/d; IVH: 49–108 mg/d). None of the subgroups achieved AA intakes that met current recommendations (Figure 5C). The mean DHA intakes were similar across the study subgroups. However, infants with NEC and IVH did not meet the recommended DHA intake of 100 mg/d at any of the investigated timepoints (NEC: 44–98 mg/d, IVH: 52–94 mg/d) (Figure 5D).

### 3.4. Macronutrient Intake in Breastfed, Formula-Fed and Mixed-Fed Infants

To investigate whether the type of milk-feeding impacts various nutrient intakes during the complementary feeding period, we performed a subgroup analysis comparing breastfed infants with formula-fed or mixed-fed infants.

**Figure 6:**
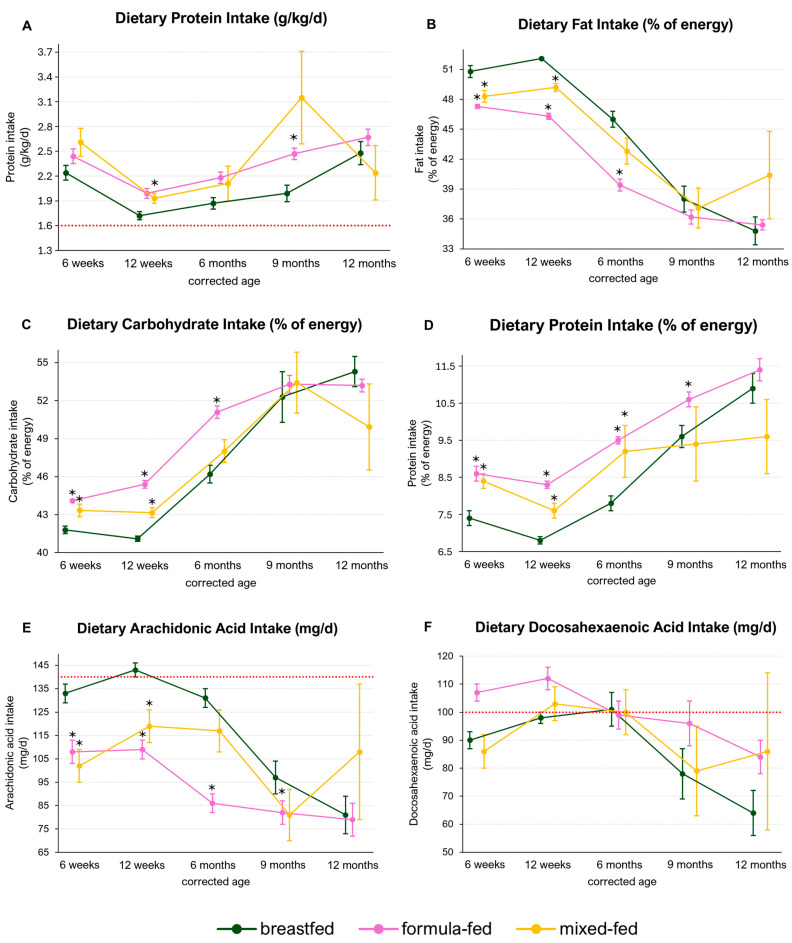
Macronutrient intake in breastfed, formula-fed and mixed-fed infants. The red lines represent current intake recommendations for the respective nutrient. Significant differences comparing breastfeeding with formula or mixed feeding (adjusted *p*-value < 0.05) are marked with *. No statistical analysis was performed at 9 and 12 months CA comparing mixed-fed with breastfed infants due to low numbers of subjects. Data are presented as the mean and standard error.

While no differences were observed for energy intake (kcal/d) between the feeding subgroups (breastfed: 506–847 kcal/d, formula: 504–814 kcal/d, mixed: 488–846 kcal/d), we found that formula- and mixed-fed infants had a higher protein intake (g/kg/d) throughout the observation period compared to breastfed infants. This difference was significant at 9 months CA between breastfed and formula-fed infants [breastfed: 1.99 g/kg/d (SE ± 0.10); formula: 2.47 g/kg/d (SE ± 0.02), *p*-adj: 0.02], and at 6 weeks CA between breastfed and mixed-fed infants [breastfed: 1.72 g/kg/d (SE ± 0.05); mixed: 1.93 g/kg/d (SE ± 0.06), *p*-adj: 0.02] (Figure 6A).

Furthermore, formula- and mixed-fed infants had a significantly higher proportional protein intake until 9 months and 6 months CA, respectively [6 weeks–9 months CA; breastfed—7.4, 6.8, 7.8, 9.6%; formula—8.6, 8.3, 9.5, 10.6%, *p*-adj: 0.001, <0.001, <0.001, 0.04; mixed—8.4, 7.6, 9.2%, *p*-adj: 0.003, <0.001. 0.04] (Figure 6B,C).

Conversely, fat intake was significantly higher in breastfed infants until 6 months and 12 weeks compared to formula- and mixed-fed infants, respectively. For fat intake, the percentages from 6 weeks until 6 months/12 weeks CA were as follows: breastfed—50.8, 52.1, 46.9%; formula—47.3, 46.3, 39.4%, *p*-adj: <0.001, <0.001, <0.001; mixed—48.3, 49.2%, *p*-adj: <0.001, <0.001. The proportional carbohydrate intake was significantly lower in breastfed compared to formula-fed infants until 6 months CA and mixed-fed infants until 12 weeks CA (6 weeks until 6 months/12 weeks CA; breastfed—41.8, 41.1, 46.2%; formula—44.1, 45.4, 51.1%, *p*-adj: <0.001, <0.001, <0.001; mixed—43.3, 43.2%, *p*-adj: 0.002, <0.001) (Figure 6D).

While DHA intake did not differ between the subgroups, breastfed infants had a significantly higher intake of AA compared to formula-fed infants until 9 months CA and compared to mixed-fed infants until 12 weeks CA (6 weeks until 9 months/12 weeks CA breastfed—133, 143, 131, 97 mg/d; formula—108, 109, 86,82 mg/d, *p*-adj: 0.001, <0.001, <0.001, 0.02; mixed—102, 119 mg/d, *p*-adj: <0.001, <0.001).

In general, dietary DHA intakes were below the recommended 100 mg/d at the beginning of the complementary feeding period and at the end of the first year of life. Recommendations for dietary AA intake (140 mg/d) were only met at 12 weeks CA in breastfed infants [1.43 mg/d (SE ± 3)] (Figure 6E,F).

## 4. Discussion

This secondary outcome analysis of a prospective, observational study in VLBW infants investigated current macronutrient intakes during the complementary feeding period, comparing early and late parent-determined introduction of solids. While some studies on nutritional intake in VLBW infants during complementary feeding exist, this is the first to present current macronutrient intakes following self-determined introduction of solids in a representative VLBW infant population.

This study found that energy and macronutrient intakes were generally unaffected by the timing of solid food introduction. However, at 12 weeks and 6 months CA, infants in the early introduction group had significantly lower proportional intakes of fat and higher proportional intakes of carbohydrates (% of energy). Dietary intake recommendations for protein, fat, LA, and ALA were met throughout the observation period, but not for AA and DHA. Comparisons between infants without comorbidities and those with BPD, NEC or IVH revealed similar nutritional intakes among the groups.

However, a subgroup analysis showed significant variation in nutritional intakes between breastfed, formula-fed, and mixed-fed infants. Formula-fed and mixed-fed infants had significantly higher intakes of protein (g/kg/d), LA, ALA, and proportional protein and carbohydrate (% of energy), whereas breastfed infants had higher mean intakes of fat (% of energy) and AA.

### 4.1. Dietary Protein Intake

The initiation of complementary feeding marks a critical period with increased susceptibility to nutritional imbalances, particularly due to changes in macronutrient composition, notably protein [16]. This vulnerability may be especially pertinent in VLBW infants due to their heightened nutritional requirements [20]. Consequently, an early introduction of protein-rich solid foods may result in an increased absolute and relative protein intake.

In this study, we observed a higher mean protein intake in the early introduction group until 6 months CA, though this difference was not statistically significant (*p*-adj = 0.20). Limited studies on protein intake during complementary feeding in VLBW infants exist, and most are outdated [10,11]. Marriott et al. found that an early introduction of solid foods in preterm infants resulted in higher energy and protein intake at 6 months CA compared to a later introduction (early: 26.7 g/d, late: 23 g/d; *p* = 0.01) [11]. Infants in the early introduction group followed a “preterm weaning strategy” with foods higher in energy and protein content. Thus, it is not unexpected that the early introduction group exhibited higher nutritional intakes and accelerated length at 12 months CA compared to the late introduction group. Additionally, that study is over 20 years old, from a time when the post-discharge nutritional management of preterm infants was different.

A recent randomized trial examined growth and nutritional intake comparing early (10–12 weeks CA) and late (16–18 weeks CA) introduction of solid foods in VLBW infants [12,13]. Infants were fed a standardized feeding regimen from solid introduction until 12 months CA. Similar to our study, mean protein intake (g/kg/d) initially declined at 12 weeks CA in both groups but increased by 6 months CA. Both studies found that mean dietary protein intakes met current recommendations (1.6 g/kg/d from 6 to 12 months of age) at all observed timepoints, suggesting that both early and late introduction of solid foods are safe for preventing undernutrition [16].

Notably, our findings indicate that proportional protein levels remained below the maximum threshold (15%) throughout the complementary feeding period (7.9–12.1% of energy) [21]. Targeting higher proportional protein levels towards the maximum threshold could potentially enhance growth and long-term health outcomes, particularly in infants experiencing growth faltering. However, the effects of varying proportional protein intakes in an infant’s diet require validation through further clinical trials, as excessive protein intake during complementary feeding may increase the risk of overweight and obesity [6,7].

### 4.2. Dietary Fat Intake

The recommended fat intake for infants aged 0–3 months is ideally set between 45 and 50% of energy, while for those aged 4–12 months, it should be maintained within 35–45% of energy [17]. We found a statistically significant difference in proportional fat intake between the groups at 3 months (early: 39.2%, late: 43.3%) and 6 months CA (early: 47.0%, late: 49.0%), with a higher intake in the late group. This difference could potentially result from the later introduction of solids itself but also from higher breastfeeding rates in the late group. Breastfed infants had higher fat intakes (% of energy) from 6 weeks until 6 months CA compared to formula-fed infants (breastfed: 46.0–50.8%, formula: 39.4–47.3%), potentially contributing to the higher fat intake levels (% of energy) noted in the late introduction group.

The findings from the ELANCE study highlight the importance of early-life fat intake for term infants, showing a negative correlation with later body fat composition [22]. A mere 1% increase in energy from fat was associated with reduced subscapular skinfold thickness, diminished fat mass, and lower serum leptin levels at 20 years of age. However, the mean proportional fat intake in the ELANCE trial was notably lower (ranging between 27.7 and 28.2% of energy at 10 months), whereas the infants in our study displayed a fat intake ranging from 35.4 to 37.6% of energy at 9 months CA. Hence, drawing a clear conclusion is not feasible regarding the clinical relevance of the observed differences in proportional fat intake between the early and late groups (4.1% at 12 weeks CA and 2% at 6 months CA) in our study. While we cannot rule out a potential clinical impact, particularly concerning the later onset of adiposity, we suggest that current intake levels appear safe regardless of the timing of solid food introduction. However, further research is needed to investigate the impact of varying fat intake proportions on clinical outcomes, with special attention to the long-term risk of obesity.

#### Dietary PUFA Intake

PUFAs, notably AA and DHA, are crucial for brain development [8]. Our investigation revealed that AA intake consistently fell below the recommended levels (140 mg/d from 0 to 6 months of life) at all examined timepoints, particularly as solid consumption increased during complementary feeding [23]. Mean DHA intakes remained within current guidelines (set at 100 mg/d until 12 months of age) until 9 months CA in the early introduction group and until 6 months CA in the late group [23]. However, at 12 months CA, mean dietary DHA intake notably lagged behind the recommended threshold in both groups, with lower intake as solid foods were introduced.

The ImNuT study by Moltu et al. showed that DHA and AA supplementation during early postnatal phases provide neuroprotective properties in VLBW infants [24]. Infants receiving 100 mg/kg AA and 50 mg/kg DHA exhibited faster linear growth and improved brain maturation compared to controls. This study shows promising results that higher PUFA intakes may improve neurodevelopmental outcome. However, the observed structural improvements remain to be tested on functional outcomes.

Though PUFA supplementation in VLBW infants has been extensively researched, high-quality trials examining PUFA intake on general health and neurodevelopmental outcomes during complementary feeding are limited. One of the very few reports on PUFA intake during complementary feeding in VLBW infants demonstrated that higher total PUFA intake, including DHA and AA, positively affected cognition and motor development at 12 months CA [25]. Although this study was a secondary outcome analysis, it highlights the critical need to improve DHA and AA intakes, as current recommendations are not being met. Enhancing PUFA intake can be achieved by incorporating PUFA-rich foods like salmon, soybean, flaxseed, or canola into the diet [26,27].

LA and ALA are essential nutrients requiring external intake as the human body cannot synthesize them [26]. Both LA and ALA did not differ between the study groups, and intake recommendations were consistently met throughout the observation period. It is well established that the nutritional adequacy of ALA and LA is not only determined by quantity but also by the ratio of LA to ALA. Previous research has shown that an imbalanced LA/ALA ratio, with heightened LA intake, significantly increases the risk of thrombosis and inflammation, contributing to obesity, metabolic syndrome, and cardiovascular disease [28,29]. Therefore, current recommendations suggest an LA/ALA ratio of 8:1 during the 0–3 month period and 7:1 from 4 to 12 months for term infants [17]. The LA/ALA ratios closely approached the recommended values in both groups. Thus, the dietary intake of LA, along with its ratio to ALA, can be considered safe for VLBW infants during the complementary feeding period, regardless of the timing of solid food introduction.

### 4.3. Comorbidities

This study represents the first attempt to assess nutritional intake during complementary feeding while specifically focusing on infants with preterm birth-related comorbidities. We found that the prevalence of infants with BPD was significantly higher in the late feeding group (early vs. late, 12% vs. 28%; *p* = 0.01), indicating that infants diagnosed with BPD were introduced to solids at a significantly later stage. The mean age at which infants with BPD started solid foods was 18.1 weeks CA [14]. Additionally, we observed a non-significant trend among parents/legal guardians of infants diagnosed with NEC to delay the introduction of solid foods compared to parents/legal guardians of infants without this comorbidity [14].

Infants with comorbidities, particularly BPD, NEC, or IVH, face an increased risk of encountering feeding difficulties, potentially leading to inadequate nutrient intakes [30]. This study demonstrated that protein and energy intakes were generally similar among infants with and without NEC, BPD, or IVH. Moreover, current recommendations for protein intake (g/kg/d) were met throughout the observation period within all subgroups. However, it remains uncertain whether these recommendations are adequate for infants with comorbidities, who may have heightened energy and nutrient requirements due to the burden of chronic illness [31]. The study by Bott et al. noted that undernutrition at two years of age in infants with BPD correlates with later childhood undernutrition, attributed not only to poor intake but also to abnormal energy balance and persistent heightened resting energy expenditure [32].

The primary outcomes of our study indicated that infants with BPD and NEC had significantly lower weight-for-age z-scores at 12 months CA compared to infants without comorbidities (weight: no comorbidities: −0.18 ± 1.21; BPD: −0.82 ± 1.52; NEC: −1.26 ± 1.06). Additionally, infants with BPD had lower length-for-age z-scores throughout the observation period, though statistically significant differences were observed only until 6 months CA (no comorbidities: 0.01 ± 1.22; BPD: −0.83 ± 1.03) [14]. Similar nutritional intakes, particularly in energy and protein among the subgroups, but lower z-scores for weight-for-age and length-for-age at the end of the first year of life in infants with BPD and NEC potentially indicate inadequate energy and nutrient intakes in infants with comorbidities. Therefore, infants with comorbidities may need different thresholds and guidelines, particularly for protein and energy intake, even during the complementary feeding period.

DHA and AA are important for brain function and were shown to improve motor and cognitive function at 12 months CA [25]. Although the dietary intakes of DHA and AA did not differ between infants with and without comorbidities, this study showed that intakes of DHA and AA were generally low, particularly in infants with NEC and IVH, not meeting intake recommendations in most of the investigated infants. Thus, our results underline the urgent need for increased intakes in infants with NEC and IVH as both conditions are associated with impaired neurodevelopment [33,34].

Overall, there remains a significant knowledge gap regarding the post-discharge dietary requirements and optimal nutrient intake for VLBW infants with comorbidities. Further analyses of dietary nutrient intakes in larger cohorts of infants with comorbidities are essential to pinpoint critical nutrients and identify potential targets to improve growth and neurodevelopmental outcomes.

### 4.4. Type of Milk Feeding

We found significant variations in nutrient intake among breastfed, formula-fed, and mixed-fed infants. While energy intake was similar across the subgroups, protein intake (g/kg/d) was notably higher in formula- and mixed-fed infants compared to breastfed infants, likely reflecting differences in infant formula composition compared to breast milk [35]. Moreover, our results indicated higher proportional fat intake and lower proportional protein and carbohydrate intake in breastfed infants compared to formula-fed infants, consistent with the previous literature [36]. The so-called “breastfeeding paradox” suggests that very preterm breastfed infants experience slower weight gain compared to formula-fed infants but show increased neurocognitive development [37]. Even though this paradox was mainly described during hospitalization, the lower intake of protein and the difference in macronutrient distribution in breastfed infants, together with a variety of bioactive human milk substances, may be a contributing factor even during the period of complementary feeding.

Interestingly, DHA levels were comparable among breastfed, mixed-fed, and formula-fed infants, whereas AA intake was significantly lower in formula- and mixed-fed infants. Human milk naturally provides both AA and DHA [38]. However, infant formula is required to include only DHA but not AA [39]. This difference may partially explain why AA intake was lower in formula-fed infants, while DHA intake was similar among the groups. Therefore, particular attention should be paid to AA intake in formula-fed infants during the complementary feeding period. Nonetheless, a balanced ratio between omega-3 and omega-6 fatty acids is of critical importance as an excessive intake of the latter is associated with inflammatory processes [28,29]. In general, mean DHA and AA intakes decreased as the amount of solid foods increased, highlighting the need to generally enhance the intake of both PUFAs through solid foods, regardless of the type of milk feeding.

While both breastfeeding and formula feeding can provide adequate nutrition for infants, significant differences in nutritional intake exist between the types of feeding. In accordance with the previous literature, these differences suggest that the type of milk feeding should influence the quality of solid foods offered to infants. Caroli et al. recommend that breastfed infants should be introduced to protein-rich foods from the beginning of the complementary feeding period such as meat, fish, legumes, and eggs [35]. In contrast, exclusively formula-fed infants should be offered a greater variety of fruits and vegetables rather than additional protein-rich foods. However, it is important to note that the recommendations by Caroli et al. specifically address the introduction of different solid foods in breastfed and formula-fed full-term infants, starting from 6 months onwards. Nevertheless, the nutritional disparities observed between breastfed and formula-fed VLBW infants suggest that these recommendations may also be applicable when solids are introduced earlier. Further research is essential to comprehensively address these differences through well-designed studies.

Infants who are discharged with suboptimal weight for their postconceptional age face a higher risk of experiencing long-term growth failure.

To support adequate nutrient intake in these infants, human milk should be supplemented with a human milk fortifier and formula-fed infants should receive enriched infant formulas, at least until a postconceptional age of 40 weeks, and potentially up to 52 weeks postconceptional age, to support optimal growth and development [40]. Our study showed that infants who received preterm infant formula, enriched infant formula or protein-enriched human milk at 6 weeks CA were introduced to solids at a median age at of 16.3 weeks CA (IQR: 13.6–19.0), whereas those who did not receive any kind of fortification were introduced to solids at a median age of 16.1 weeks CA (IQR: 13.3–19.0); *p* = 0.94. Although limited information exists on the timing of solid food introduction in infants with growth failure, this study provides first evidence that while fortification may support an infant’s growth post-discharge, it does not inherently delay the introduction of solid foods. However, this study was not powered to detect a difference in the introduction of solids in infants with or without fortification post-discharge. Hence, further research is crucial to explore both the timing of solid food introduction and the nutritional intake during complementary feeding in infants experiencing growth faltering.

### 4.5. Strengths and Limitations

This secondary analysis of a prospective observational trial provides important insights into complementary feeding practices among VLBW infants, enhancing our understanding of nutritional intakes during the critical first year of life.

Additionally, the inclusion of a diverse collective of VLBW infants with comorbidities enhances the generalizability of the findings. The study’s limitations are highlighted by its reliance on average breast milk intake data and the fact that the study was not powered to detect a difference in nutritional intakes between the study groups. Additionally, this secondary outcome analysis is constrained by the low number of valid dietary records for analysis at 9 and 12 months CA.

Overall, while this study offers valuable contributions to our understanding of complementary feeding practices in a representative preterm cohort, it also highlights areas for further research and improvement in study design to address limitations and enhance the validity and reliability of findings.

## 5. Conclusions

This secondary analysis of a prospective, observational study examined nutrient intake during self-directed complementary feeding in a representative cohort of VLBW infants. The findings indicate that both absolute and relative protein, as well as proportional fat intake, align with current recommendations. Therefore, macronutrient intake during complementary feeding can be considered safe, irrespective of the timing of solid food introduction in this vulnerable population. However, further research is crucial to explore variations in proportional macronutrient intakes and their potential impact on long-term health outcomes.

This study found that infants with comorbidities had similar macronutrient levels compared to those without. However, it remains unclear whether the current energy and protein intakes are adequate for these infants. Therefore, further research is essential to establish reference values for this subpopulation of VLBW infants to optimize health outcomes. Additionally, it is essential to highlight the importance of increasing DHA and AA intakes to enhance neurodevelopmental outcomes in VLBW infants, particularly those with comorbidities, as current intake levels often do not meet recommended thresholds.

## Figures and Tables

**Table 1:** Dietary protocols valid for analysis.

CA	Early Group *n* = 115	Late Group *n* = 82	Total
6 weeks	86 (74.8%)	60 (73.2%)	146
12 weeks	106 (92.2%)	64 (78.0%)	170
6 months	90 (78.2%)	66 (80.5%)	156
9 months	63 (54.7%)	51 (62.2%)	114
12 months	65 (56.0%)	57 (69.5%)	122

CA: corrected age.

**Table 2:** Baseline characteristics and nutrition.

Parameter	Early (*n* = 115)	Late (*n* = 82)	*p*-Value
Neonatal parameters			
Male sex	69 (60%)	36 (44%)	**0.04**
Gestational age (weeks)	27 + 1 (25 + 3–28 + 3)	26 + 3 (24 + 6–28 + 2)	0.33
Birth weight (g)	925 (718–1133)	820 (650–1078)	0.13
Small for gestational age	4 (3.5%)	4 (4.9%)	0.62
Neonatal morbidities			
Necrotizing enterocolitis ≥ grade II	5 (4%)	6 (7%)	0.42
Bronchopulmonary dysplasia	14 (12%)	23 (28%)	**0.01**
Intraventricular hemorrhage ≥ grade II	17 (15%)	12 (15%)	0.74
Periventricular leukomalacia	0 (0%)	1 (1%)	/
Nutrition			
Introduction of solid foods (weeks CA)	13.7 (12.6–15.4)	19.4 (18.3–21.7)	**<0.001**
Type of first solid food			
Vegetables	82 (71%)	54 (66%)	0.69
Fruits	17 (15%)	14 (17%)	0.82
Milk and dairy products	0 (0%)	1 (1%)	/
Cereals (with gluten)	1 (1%)	1 (1%)	/
Cereals (without gluten)	1 (1%)	2 (2%)	/
Several	11 (10%)	7 (9%)	0.99
Type of milk feeding at 6 weeks CA			
Breastfed	28 (24%)	34 (42%)	**0.01**
Formula fed	66 (57%)	31 (38%)	**0.01**
Mixed fed	18 (16%)	15 (18%)	1.00

Categorical data are presented as numbers with percentages in parentheses. Continuous data are presented as the median and interquartile range in parentheses. CA: corrected age; *p*-values < 0.05 were considered statistically significant and marked bold.

## Data Availability

The study protocol and the individual participant data that underlie the results reported in this article, after de-identification, are available upon request from the corresponding author 6 months after publication. Researchers will need to state the aims of any analyses and provide a methodologically sound proposal. Proposals should be directed to nadja.haiden@kepleruniklinikum.at. Data requestors will need to sign a data access agreement and, in keeping with patient consent for secondary use, obtain ethical approval for any new analyses due to ethical reasons.

## Supplementary Materials

The following supporting information can be downloaded at: https://www.mdpi.com/article/10.3390/nu16193422/s1, Supplementary Table S1: Dietary macronutrient intake comparing early and late introduction of complementary feeding; Supplementary Table S2: Number of infants with neonatal comorbidities; Supplementary Table S3: Nutritional intake in infants without comorbidities and infants with BPD, NEC, or IVH; Supplementary Table S4: Number of available dietary records from infants with/without bronchopulmonary dysplasia; Supplementary Table S5: Nutrient intake comparing early and late introduction of solids in infants with bronchopulmonary dysplasia; Supplementary Table S6: Number of available dietary records from infants with/without necrotizing enterocolitis ≥ grade II; Supplementary Table S7: Nutrient intake comparing early and late introduction of solids in infants with necrotizing enterocolitis ≥ grade II; Supplementary Table S8: Number of available dietary records from infants with/without intraventricular hemorrhage ≥ grade II; Supplementary Table S9: Nutrient intake comparing early and late introduction of solids in infants with intraventricular hemorrhage ≥ grade II; Supplementary Table S10: Nutrient intake comparing breastfed, formula-fed, mixed-fed and no milk intake; Supplementary Table S11: Number of protocols according to type of feeding in the early and late group; Supplementary Table S12: Overview of different formulas used in this observational study; Supplementary Table S13: Breastfed infants that received fortification. Supplementary Table S14: Range of nutrients of infant formulas that were additionally entered into the nutritional software due to missing data or changes in formulation over time; Supplementary Table S15: Nutrient intake comparing early and late introduction of solid foods in breastfed, formula-fed, mixed-fed infants and without milk-feeding.
