# Supplementary material for: Macronutrient Intake during Complementary Feeding in Very Low Birth Weight Infants Comparing Early and Late Introduction of Solid Foods: A Secondary Outcome Analysis
Source: Nutrients. 2024 Oct 9;16(19):3422. doi: 10.3390/nu16193422 (PMC11478545; doi:10.3390/nu16193422)
Supplement: Supplementary file 1 [file nutrients-16-03422-s001.zip › nutrients-3207554-supplementary.pdf]

Supplementary Materials

Macronutrient Intake during Complementary Feeding in Very Low Birth Weight Infants comparing Early and Late Introduction of Solid Foods: A Secondary Outcome Analysis

1. Macronutrient intake comparing early and late introduction of solid foods

Nutrient intake was evaluated by comparing early and late introduction of solid foods using linear mixed-effects models. These models included the complementary feeding group, sex, gestational age, and nutrition at 6 weeks corrected age (CA) as covariates, with a random intercept to address potential correlation among siblings of multiple births. Marginal means, calculated from these models and averaged across covariates, were determined for the two groups. Standard errors and p-values were then calculated to test the null hypothesis of no difference between the groups. The p-values for between-group comparisons of the same nutrient at different timepoints were adjusted using the Bonferroni-Holm method. Statistical significance was set at p-adj. < 0.05.

Dietary intakes were categorized into two main types: solids and milk (breastmilk, infant formula). The distribution of these sources is presented as percentage of the total nutritional intake. Tea, water, and liquid beverages were collectively categorized as solids, despite liquids not being considered in determining the beginning of complementary feeding.

Dietary intakes for fat and carbohydrates are only presented in % of energy as no other reference values for infants during complementary feeding are available.

Supplementary Table S1: *Dietary macronutrient intake comparing early and late introduction of complementary feeding.*

Protein (g/kg/d)

CA	Early Mean ± SE	Early % (milk)	Early % (solids)	Late Mean ± SE	Late % (milk)	Late % (solids)	p-adj.
**6 weeks**	2.52 (±0.11)	99.9	0.1	2.31 (±0.11)	100.0	0.0	0.38
**12 weeks**	1.95 (±0.06)	95.6	4.4	1.88 (±0.07)	100.0	0.0	0.77
**6 months**	2.20 (±0.08)	56.6	43.4	1.98 (±0.09)	69.9	30.1	0.38
**9 months**	2.47 (±0.11)	34.3	65.7	2.51 (±0.12)	37.6	62.4	0.95
**12 months**	2.71 (±0.12)	26.6	73.4	2.72 (±0.13)	25.0	75.0	0.95

Recommended daily protein intake: 1.6 g/kg/d.

Energy (kcal/d)

CA	Early Mean ± SE	Early % (milk)	Early % (solids)	Late Mean ± SE	Late % (milk)	Late % (solids)	p-adj.
**6 weeks**	514 (±14)	99.9	0.1	484 (±14)	100.0	0.0	0.23
**12 weeks**	535 (±12)	96.2	3.8	510 (±13)	100.0	0.0	0.23
**6 months**	655 (±16)	64.9	35.1	594 (±17)	76.5	23.5	0.05
**9 months**	741 (±21)	42.6	57.4	738 (±22)	46.4	53.6	0.92
**12 months**	791 (±26)	32.8	67.2	815 (±26)	32.6	67.4	0.64

Recommended daily energy intake: 0-3 months: 500-550 kcal/d; 4-12 months: 600-700 kcal/d.

Protein (% of energy)

CA	Early Mean ± SE	Early % (milk)	Early % (solids)	Late Mean ± SE	Late % (milk)	Late % (solids)	p-adj.
**6 weeks**	8.2 (±0.21)	99.9	0.1	8.0 (±0.21)	100.0	0.0	0.60
**12 weeks**	7.9 (±0.13)	95.6	4.4	7.8 (±0.16)	100.0	0.0	0.60
**6 months**	9.3 (±0.24)	56.6	43.4	8.8 (±0.26)	69.9	30.1	0.43
**9 months**	10.7 (±0.27)	34.3	65.7	10.4 (±0.28)	37.6	62.4	0.60
**12 months**	12.3 (±0.34)	26.6	73.4	11.3 (±0.36)	25.0	75.0	0.25

Recommended daily protein intake: <15% of energy.

Fat (% of energy)

CA	Early Mean ± SE	Early % (milk)	Early % (solids)	Late Mean ± SE	Late % (milk)	Late % (solids)	p-adj.
**6 weeks**	48.6 (±0.37)	100	0	49.0 (±0.36)	100	0	0.38
**12 weeks**	47.5 (±0.38)	98.3	1.7	49.3 (±0.43)	100	0	**0.005**
**6 months**	39.7 (±0.68)	77.7	22.3	43.5 (±0.75)	84.7	15.3	**0.001**
**9 months**	35.4 (±0.88)	54.2	45.8	37.6 (±0.92)	58.2	41.8	0.38
**12 months**	35.8 (±0.74)	40.5	59.5	34.7 (±0.75)	42.2	57.8	0.38

Recommended daily fat intake 0-3 months: 45-50% of energy; 4-12 months: 35-45% of energy.

Carbohydrates (% of energy)

CA	Early Mean ± SE	Early % (milk)	Early % (solids)	Late Mean ± SE	Late % (milk)	Late % (solids)	p-adj.
**6 weeks**	43.2 (±0.28)	99.9	0.1	43.0 (±0.28)	100	0	0.59
**12 weeks**	44.5 (±0.35)	94.6	5.4	42.9 (±0.39)	100	0	**0.003**
**6 months**	51.1 (±0.61)	57.7	42.3	47.7 (±0.67)	71.3	28.6	**0.002**
**9 months**	54.0 (±0.83)	36.9	63.1	52.0 (±0.88)	40.6	59.4	0.14
**12 months**	51.9 (±0.73)	29.5	70.5	54.0 (±0.74)	27.9	72.1	0.06

/

Arachidonic Acid (mg/d)

CA	Early Mean ± SE	Early % (milk)	Early % (solids)	Late Mean ± SE	Late % (milk)	Late % (solids)	p-adj.
**6 weeks**	114.0 (±5.3)	99.9	0.1	113.0 (±5.3)	100.0	0.0	0.94
**12 weeks**	108.0 (±4.7)	95.6	4.4	121.0 (±5.3)	100.0	0.0	0.13
**6 months**	89.1 (±5.1)	56.6	43.4	103.4 (±5.5)	69.9	30.1	0.13
**9 months**	83.6 (±7.9)	34.3	65.7	75.2 (±8.2)	37.6	62.4	0.59
**12 months**	74.1 (±7.6)	26.6	73.4	66.5 (±7.7)	25.0	75.0	0.59

Recommended daily AA intake: 0-6 months: 140 mg/d; 6-12 months: no recommendations exist.

Docosahexaenoic Acid (mg/d)

CA	Early Mean ± SE	Early % (milk)	Early % (solids)	Late Mean ± SE	Late % (milk)	Late % (solids)	p-adj.
**6 weeks**	94.8 (±4.4)	100.0	0.0	94.1 (±4.4)	100.0	0.0	0.91
**12 weeks**	97.7 (±4.2)	97.8	2.2	105.0 (±4.8)	100.0	0.0	0.60
**6 months**	95.5 (±5.7)	83.7	16.3	98.1 (±6.2)	91.4	8.6	0.91
**9 months**	86.8 (±9.3)	65.1	34.9	78.1 (±9.8)	72.0	28.0	0.85
**12 months**	81.4 (±7.8)	50.5	49.5	66.7 (±7.9)	61.1	38.9	0.60

Recommended daily DHA intake: 0-12 months: 100 mg/d.

Linoleic Acid (% of energy)

CA	Early Mean ± SE	Early % (milk)	Early % (solids)	Late Mean ± SE	Late % (milk)	Late % (solids)	p-adj.
**6 weeks**	6.0 (±0.11)	100.0	0.0	6.0 (±0.11)	100.0	0.0	0.98
**12 weeks**	6.2 (±0.10)	97.1	2.9	6.0 (±0.11)	100.0	0.0	0.52
**6 months**	5.6 (±0.13)	72.4	27.6	5.7 (±0.14)	82.0	18.0	0.55
**9 months**	5.0 (±0.16)	51.5	48.5	5.0 (±0.17)	54.8	45.2	0.98
**12 months**	5.0 (±0.17)	41.2	58.8	4.7 (±0.17)	40.7	59.3	0.53

Recommended daily LA intake: 0-3 months: 4.0% of energy; 4-12 months: 3.5% of energy.

α-Linolenic Acid (% of energy)

CA	Early Mean ± SE	Early % (milk)	Early % (solids)	Late Mean ± SE	Late % (milk)	Late % (solids)	p-adj.
**6 weeks**	0.71 (±0.02)	100.0	0.0	0.66 (±0.03)	100.0	0.0	0.25
**12 weeks**	0.75 (±0.02)	95.1	4.9	0.70 (±0.03)	100.0	0.0	0.25
**6 months**	0.86 (±0.05)	61.2	23.8	0.82 (±0.05)	72.2	27.8	0.44
**9 months**	0.87 (±0.08)	42.9	57.1	0.78 (±0.08)	44.5	55.5	0.44
**12 months**	0.77 (±0.04)	35.0	65.0	0.67 (±0.04)	35.5	64.5	0.25

Recommended daily ALA intake: 0-12 months: 0.5 % of energy.

LA/ALA ratio

CA	Early Mean ± SE	Early % (milk)	Early % (solids)	Late Mean ± SE	Late % (milk)	Late % (solids)	p-adj.
**6 weeks**	9.1 (±0.2)	/	/	9.6 (±0.2)	/	/	0.23
**12 weeks**	8.7 (±0.2)	/	/	9.2 (±0.2)	/	/	0.23
**6 months**	7.0 (±0.3)	/	/	7.7 (±0.3)	/	/	0.23
**9 months**	6.8 (±0.3)	/	/	6.8 (±0.4)	/	/	0.85
**12 months**	7.3 (±0.3)	/	/	7.5 (±0.3)	/	/	0.78

Recommended daily LA/ALA ratio: 0-3 months: 8:1; 4-12 months: 7:1.

CA: corrected age; SE: standard error. p-adj. <0.05 were considered statistically significant and marked bold.

2. Subgroup analysis: macronutrient intake in infants with and without comorbidities

Supplementary Table S2: *Number of infants with neonatal comorbidities.*

Parameter	Early (n=115)	Late (n=82)
Necrotizing enterocolitis ≥ grade II	5 (4%)	6 (7%)
Bronchopulmonary dysplasia	14 (12%)	23 (28%)
Intraventricular haemorrhage ≥ grade II	17 (15%)	12 (15%)
Periventricular leukomalacia	0 (0%)	1 (1%)

2.1. Subgroup analysis of nutritional intake in infants without comorbidities and infants with BPD, NEC or IVH

Nutrient intake was evaluated by comparing infants without comorbidities and infants diagnosed with bronchopulmonary dysplasia (BPD), necrotizing enterocolitis ≥ grade II (NEC), or intraventricular hemorrhage ≥ grade II (IVH). To detect differences between study subgroups, students t-test or Mann-Whitney-U-test were applied. As an additional analysis, the p-values for between-subgroup comparisons of the same nutrient at different timepoints were adjusted using the Bonferroni-Holm method. Standard errors and adjusted p-values were calculated to test the null hypothesis of no difference between the groups. Statistical significance was set at p-adj. < 0.05. The statistical analysis was performed using R version 4.1.1 (R Core Team, 2022).

Supplementary Table S3: *Nutritional intake in infants without comorbidities and infants with BPD, NEC or IVH.*

CA	Protein (g/kg/d)						
	Infants without BPD, NEC, IVH<br>Mean ± SE	BPD<br>Mean ± SE	p-adj.	NEC<br>Mean ± SE	p-adj.	IVH<br>Mean ± SE	p-adj.
**6 weeks**	2.36 (±0.08)	2.55 (±0.19)	0.84	2.87 (±0.25)	0.41	2.44 (±0.07)	0.94
**12 weeks**	1.91 (±0.04)	1.97 (±0.11)	0.93	2.10 (±0.15)	0.51	1.89 (±0.17)	0.57
**6 months**	2.15 (±0.08)	1.93 (±0.10)	0.51	2.27 (±0.26)	0.84	1.95 (±0.11)	0.84
**9 months**	2.46 (±0.10)	2.40 (±0.10)	0.91	2.21 (±0.20)	0.84	2.25 (±0.15)	0.57
**12 months**	2.75 (±0.09)	2.71 (±0.21)	0.84	2.28 (±0.21)	0.41	2.44 (±0.21)	0.41

Recommended daily protein intake: 1.6 g/kg/d.

CA	Energy (kcal/d)						
	Infants without BPD, NEC, IVH<br>Mean ± SE	BPD<br>Mean ± SE	p-adj.	NEC<br>Mean ± SE	p-adj.	IVH<br>Mean ± SE	p-adj.
**6 weeks**	508 (±9)	489 (±24)	0.42	465 (±29)	0.32	482 (±28)	0.42
**12 weeks**	546 (±9)	513 (±18)	0.08	470 (±26)	0.08	505 (±25)	0.08
**6 months**	646 (±13)	614 (±27)	0.30	634 (±55)	0.70	608 (±25)	0.32

CA	Infants without BPD, NEC, IVH Mean ± SE	BPD Mean ± SE	p-adj.	NEC Mean ± SE	p-adj.	IVH Mean ± SE	p-adj.
**9 months**	753 (±17)	730 (±24)	0.65	682 (±63)	0.30	693 (±39)	0.14
**12 months**	821(±18)	784 (±35)	0.41	706 (±45)	0.13	739 (±54)	0.08

Recommended daily energy intake: 0-3 months: 500-550 kcal/d; 4-12 months: 600-700 kcal/d.

CA	Protein (% energy)						
	Infants without BPD, NEC, IVH Mean ± SE	BPD Mean ± SE	p-adj.	NEC Mean ± SE	p-adj.	IVH Mean ± SE	p-adj.
**6 weeks**	8.2 (±0.1)	8.7 (±0.4)	0.91	9.5 (±0.6)	0.22	8.3 (±0.4)	0.85
**12 weeks**	7.8 (±0.1)	7.9 (±0.2)	0.91	8.8 (±0.5)	0.22	8.0 (±0.3)	0.91
**6 months**	9.3 (±0.2)	8.6 (±0.3)	0.33	8.9 (±0.5)	0.85	8.4 (±0.3)	0.22
**9 months**	10.7 (±0.2)	9.9 (±0.4)	0.18	10.1 (±1.0)	0.27	10.0 (±0.4)	0.39
**12 months**	12.3 (±0.3)	11.2 (±0.5)	0.27	10.3 (±0.3)	0.18	11.0 (±0.5)	0.22

Recommended daily protein intake: <15% of energy.

CA	Fat (% of energy)						
	Infants without BPD, NEC, IVH Mean ± SE	BPD Mean ± SE	p-adj.	NEC Mean ± SE	p-adj.	IVH Mean ± SE	p-adj.
**6 weeks**	48.2 (±0.3)	48.9 (±0.5)	0.61	47.3 (±1.0)	0.61	48.0 (±0.7)	0.96
**12 weeks**	47.9 (±0.3)	48.4 (±0.6)	0.61	47.9 (±0.9)	0.96	48.0 (±1.0)	0.61
**6 months**	40.8 (±0.6)	42.7 (±1.2)	0.61	41.3 (±1.9)	0.96	40.8 (±1.4)	0.98
**9 months**	35.9 (±0.7)	37.1 (±1.3)	0.66	38.9 (±2.6)	0.61	37.7 (±2.0)	0.61
**12 months**	34.9 (±0.6)	34.3 (±1.2)	0.94	38.0 (±2.4)	0.61	35.2 (±1.4)	0.96

Recommended daily fat intake 0-3 months: 45-50% of energy; 4-12 months: 35-45% of energy.

CA	Carbohydrates (% of energy)						
	Infants without BPD, NEC, IVH Mean ± SE	BPD Mean ± SE	p-adj.	NEC Mean ± SE	p-adj.	IVH Mean ± SE	p-adj.
**6 weeks**	43.6 (±0.2)	42.5 (±0.4)	0.09	43.2 (±0.8)	0.80	43.7 (±0.6)	0.85
**12 weeks**	44.3 (±0.3)	43.7 (±0.5)	0.69	43.3 (±0.7)	0.69	43.9 (±1.0)	0.69
**6 months**	49.9 (±0.5)	48.7 (±1.1)	0.69	49.7 (±1.9)	0.84	50.8 (±1.3)	0.80
**9 months**	53.4 (±0.6)	53.0 (±1.1)	0.84	51.0 (±1.7)	0.75	52.1 (±1.7)	0.80
**12 months**	52.9 (±0.6)	54.5 (±1.1)	0.69	51.7 (±2.3)	0.80	53.8 (±1.3)	0.80

/

CA	Arachidonic Acid (mg/d)						
	Infants without BPD, NEC, IVH Mean ± SE	BPD Mean ± SE	p-adj.	NEC Mean ± SE	p-adj.	IVH Mean ± SE	p-adj.
**6 weeks**	117 (±4)	102 (±9)	0.43	98 (±14)	0.44	101 (±7)	0.17
**12 weeks**	122 (±4)	112 (±7)	0.48	99 (±17)	0.44	108 (±11)	0.44
**6 months**	101 (±4)	102 (±9)	0.88	93 (±19)	0.67	84 (±9)	0.38
**9 months**	82 (±6)	81 (±9)	0.66	59 (±27)	0.44	76 (±13)	0.85
**12 months**	80 (±7)	65 (±10)	0.52	43 (±14)	0.17	49 (±10)	0.17

Recommended daily AA intake: 0-6 months: 140 mg/d; 6-12 months: no recommendations exist.

CA	Docosahexaenoic Acid (mg/d)						
	Infants without BPD, NEC, IVH Mean ± SE	BPD Mean ± SE	p-adj.	NEC Mean ± SE	p-adj.	IVH Mean ± SE	p-adj.
**6 weeks**	103 (±3)	93 (±6)	0.30	89 (±7)	0.33	93 (±8)	0.34
**12 weeks**	110 (±3)	104 (±6)	0.28	98 (±18)	0.28	94 (±10)	0.26
**6 months**	105 (±5)	89 (±7)	0.33	95 (±12)	0.65	80 (±8)	0.14

CA							
**9 months**	90 (±8)	101 (±14)	0.34	53 (±24)	0.26	92 (±21)	0.79
**12 months**	82 (±7)	65 (±8)	0.44	44 (±17)	0.14	52 (±9)	0.14

Recommended daily DHA intake: 0-12 months: 100 mg/d.

CA	Linoleic Acid (% of energy)						
	**Infants without BPD, NEC, IVH**	**BPD**	**p-adj.**	**NEC**	**p-adj.**	**IVH**	**p-adj.**
	**Mean ± SE**	**Mean ± SE**		**Mean ± SE**		**Mean ± SE**	
**6 weeks**	6.14 (±0.09)	6.44 (±0.19)	0.77	6.09 (±0.41)	0.98	6.19 (±0.21)	0.77
**12 weeks**	6.19 (±0.08)	6.37 (±0.15)	0.77	6.07 (±0.40)	0.77	6.03 (±0.24)	0.77
**6 months**	5.55 (±0.09)	5.76 (±0.21)	0.77	5.90 (±0.39)	0.77	5.54 (±0.33)	0.46
**9 months**	5.04 (±0.12)	4.92 (±0.21)	0.77	4.96 (±1.00)	0.92	4.89 (±0.39)	0.77
**12 months**	4.81 (±0.11)	4.65 (±0.22)	0.77	5.24 (±0.64)	0.77	4.71 (±0.39)	0.85

Recommended daily LA intake: 0-3 months: 4.0% of energy; 4-12 months: 3.5% of energy.

CA	α-Linolenic Acid (% of energy)						
	**Infants without BPD, NEC, IVH**	**BPD**	**p-adj.**	**NEC**	**p-adj.**	**IVH**	**p-adj.**
	**Mean ± SE**	**Mean ± SE**		**Mean ± SE**		**Mean ± SE**	
**6 weeks**	0.72 (±0.02)	0.76 (±0.04)	0.83	0.74 (±0.11)	0.94	0.79 (±0.07)	0.92
**12 weeks**	0.72 (±0.02)	0.79 (±0.04)	0.83	0.71 (±0.08)	0.94	0.75 (±0.06)	0.94
**6 months**	0.83 (±0.04)	0.81 (±0.06)	0.94	0.96 (±0.12)	0.83	0.83 (±0.07)	0.94
**9 months**	0.81 (±0.04)	0.74 (±0.04)	0.94	0.87 (±0.09)	0.83	1.01 (±0.31)	0.94
**12 months**	0.74 (±0.03)	0.70 (±0.04)	0.94	0.92 (±0.10)	0.56	0.72 (±0.05)	0.94

Recommended daily ALA intake: 0-12 months: 0.5 % of energy.

CA	LA/ALA ratio						
	**Infants without BPD, NEC, IVH**	**BPD**	**p-adj.**	**NEC**	**p-adj.**	**IVH**	**p-adj.**
	**Mean ± SE**	**Mean ± SE**		**Mean ± SE**		**Mean ± SE**	
**6 weeks**	9.1 (±0.2)	9.1 (±0.4)	0.94	9.3 (±0.9)	0. 94	8.6 (±0.6)	0. 94
**12 weeks**	8.8 (±0.2)	8.6 (±0.4)	0. 94	9.2 (±0.6)	0. 94	8.7 (±0.7)	0. 94
**6 months**	7.4 (±0.2)	7.6 (±0.4)	0. 94	6.8 (±0.7)	0. 94	7.2 (±0.5)	0. 94
**9 months**	6.8 (±0.2)	7.2 (±0.6)	0. 94	5.4 (±0.8)	0. 94	6.7 (±0.7)	0. 94
**12 months**	7.2 (±0.3)	7.0 (±0.4)	0. 94	6.0 (±0.6)	0. 94	6.8 (±0.4)	0. 94

Recommended daily LA/ALA ratio: 0-3 months: 8:1; 4-12 months: 7:1.

CA: corrected age; BPD: bronchopulmonary dysplasia; NEC: necrotizing enterocolitis;, IVH: intraventricular hemorrhage; AA: arachidonic acid; DHA: docosahexaenoic acid; LA: linoleic acid; ALA: α-linolenic acid; SE: standard error. p-adj. <0.05 were considered statistically

2.2. Subgroup analysis: Macronutrient intake comparing early and late introduction of solid foods in infants with and without comorbidities

Nutrient intake was evaluated by comparing the timepoint of solid food introduction (early vs. late) in infants without comorbidities and comorbidities and infants diagnosed with bronchopulmonary dysplasia (BPD), necrotizing enterocolitis ≥ grade II (NEC), or intraventricular hemorrhage ≥ grade II (IVH). To detect differences between study subgroups, students t-test or Mann-Whitney-U-test were applied. For infants with NEC no statistical testing was conducted between the early and late group due to the low number of infants with NEC. Standard errors and adjusted p-values were calculated to test the null hypothesis of no difference between the groups. Statistical significance was set at p-adj. < 0.05. As an additional analysis, the p-values for between-subgroup comparisons of the same nutrient at different timepoints were adjusted using the Bonferroni-Holm method. The statistical analysis was performed using R version 4.1.1 (R Core Team, 2022).

2.2.1. Macronutrient intake comparing early and late introduction of solids in infants with BPD

Supplementary Table S4: *Numbers of available dietary records from infants with/without bronchopulmonary dysplasia.*

CA	EARLY			LATE		
	total	Infants without BPD, NEC, IVH	BPD	total	Infants without BPD, NEC, IVH	BPD
**6 weeks**	75	64 (85%)	11 (15%)	54	37 (69%)	17 (31%)
**12 weeks**	92	79 (86%)	13 (14%)	57	36 (63%)	21 (37%)
**6 months**	77	66 (86%)	11 (14%)	58	41 (71%)	17 (29%)
**9 months**	54	48 (89%)	6 (11%)	46	29 (65%)	16 (35%)
**12 months**	53	48 (91%)	5 (9%)	49	32 (65%)	17 (35%)

CA: corrected age; BPD: bronchopulmonary dysplasia; NEC: necrotizing enterocolitis; IVH: intraventricular hemorrhage. Data are presented as numbers with percentages in parentheses.

Supplementary Table S5: *Nutrient intake comparing early and late introduction of solids in infants with bronchopulmonary dysplasia.*

CA	Protein (g/kg/d)			Energy (kcal/d)		
	Early Mean ± SE	Late Mean ± SE	p-adj.	Early Mean ± SE	Late Mean ± SE	p-adj.
**6 weeks**	2.82 (±0.38)	2.37 (±0.19)	0.38	511 (±48)	474 (±25)	0.57
**12 weeks**	2.18 (±0.26)	1.83 (±0.08)	0.38	546 (±34)	493 (±19)	0.57
**6 months**	2.14 (±0.19)	1.80 (±0.10)	0.35	673 (±54)	576 (±26)	0.43
**9 months**	2.65 (±0.10)	2.30 (±0.13)	0.35	721 (±47)	734 (±28)	0.81

CA	Early Mean ± SE	Late Mean ± SE	p-adj.	Early Mean ± SE	Late Mean ± SE	p-adj.
**12 months**	3.09 (±0.72)	2.59 (±0.19)	0.38	812 (±66)	775 (±42)	0.57

**CA**	**Protein (% of energy)**			**Fat (% of energy)**		
	**Early Mean ± SE**	**Late Mean ± SE**	**p-adj.**	**Early Mean ± SE**	**Late Mean ± SE**	**p-adj.**
**6 weeks**	9.0 (±0.6)	8.5 (±0.6)	0.68	48.3 (±0.8)	49.2 (±0.7)	0.45
**12 weeks**	8.3 (±0.4)	7.7 (±0.3)	0.68	47.1 (±1.1)	49.1 (±0.5)	0.45
**6 months**	8.7 (±0.4)	8.5 (±0.3)	0.68	39.5 (±1.7)	44.8 (±1.4)	0.11
**9 months**	11.3 (±0.8)	9.3 (±0.3)	0.08	35.3 (±3.6)	37.8 (±1.2)	0.45
**12 months**	11.7 (±1.6)	11.0 (±0.4)	0.68	34.2 (±3.9)	34.3 (±1.1)	0.45

**CA**	**Carbohydrates (% of energy)**			**Arachidonic Acid (mg/d)**		
	**Early Mean ± SE**	**Late Mean ± SE**	**p-adj.**	**Early Mean ± SE**	**Late Mean ± SE**	**p-adj.**
**6 weeks**	42.7 (±0.6)	42.3 (±0.5)	0.85	103 (±14)	102 (±13)	0.96
**12 weeks**	44.6 (±1.2)	43.2 (±0.4)	0.85	109 (±13)	113 (±9)	0.95
**6 months**	51.8 (±1.7)	46.7 (±1.3)	0.12	93 (±17)	109 (±9)	0.94
**9 months**	53.4 (±3.3)	52.9 (±1.0)	0.85	67 (±21)	87 (±10)	0.94
**12 months**	54.1 (±4.0)	54.6 (±1.0)	0.85	73 (±31)	63 (±9)	0.95

**CA**	**Docosahexaenoic Acid (mg/d)**			**Linoleic Acid (% of energy)**		
	**Early Mean ± SE**	**Late Mean ± SE**	**p-adj.**	**Early Mean ± SE**	**Late Mean ± SE**	**p-adj.**
**6 weeks**	97 (±10)	90 (±8)	0.71	6.5 (±0.3)	6.4 (±0.2)	0.44
**12 weeks**	113 (±11)	98 (±7)	0.71	6.4 (±0.3)	6.3 (±0.2)	0.44
**6 months**	86 (±15)	91 (±6)	0.71	5.7 (±0.5)	5.8 (±0.2)	0.87
**9 months**	106 (±49)	99 (±11)	0.71	4.5 (±0.5)	5.1 (±0.2)	0.44
**12 months**	56 (±25)	68 (±9)	0.71	4.6 (±6)	4.7 (±6)	0.44

**CA**	**α-Linolenic Acid (% of energy)**			**LA/ALA ratio (% of energy)**		
	**Early Mean ± SE**	**Late Mean ± SE**	**p-adj.**	**Early Mean ± SE**	**Late Mean ± SE**	**p-adj.**
**6 weeks**	0.82 (±0.07)	0.71 (±0.05)	0.48	8.4 (±0.6)	9.6 (±0.5)	0.73
**12 weeks**	0.86 (±0.07)	0.76 (±0.05)	0.48	8.1 (±0.6)	8.9 (±0.4)	0.73
**6 months**	0.80 (±0.08)	0.83 (±0.08)	0.98	7.4 (±0.6)	7.7 (±0.5)	0.73
**9 months**	0.66 (±0.10)	0.77 (±0.05)	0.98	8.0 (±1.8)	6.9 (±0.4)	0.73
**12 months**	0.77 (±0.12)	0.68 (±0.04)	0.98	6.4 (±0.9)	7.2 (±0.5)	0.73

CA: corrected age; SE: standard error. p-adj. <0.05 were considered statistically significant and marked bold.

2.2.2. Macronutrient intake comparing early and late introduction of solids in infants with NEC

Supplementary Table S6: *Numbers of available dietary records from infants with/without necrotizing enterocolitis ≥ grade II.*

CA	EARLY			LATE		
	total	Infants without BPD, NEC, IVH	NEC	total	Infants without BPD, NEC, IVH	NEC
**6 weeks**	67	64 (85%)	3 (5%)	43	37 (86%)	6 (14%)
**12 weeks**	84	79 (94%)	5 (6%)	42	36 (86%)	6 (14%)
**6 months**	70	66 (94%)	4 (6%)	47	41 (85%)	6 (15%)
**9 months**	50	48 (96%)	2 (4%)	33	29 (88%)	4 (12%)
**12 months**	52	48 (92%)	4 (8%)	37	32 (86%)	5 (14%)

CA: corrected age; BPD: bronchopulmonary dysplasia; NEC: necrotizing enterocolitis; IVH: intraventricular hemorrhage. Data are presented as numbers with percentages in parentheses

Supplementary Table S7: *Nutrient intake comparing early and late introduction of solids in infants with necrotizing enterocolitis ≥ grade II.*

CA	Protein (g/kg/d)		Energy (kcal/d)	
	Early	Late	Early	Late
	Mean ± SE	Mean ± SE	Mean ± SE	Mean ± SE
**6 weeks**	2.50 (±0.54)	3.05 (±0.27)	433 (±19)	481 (±42)
**12 weeks**	2.00 (±0.27)	2.17 (±0.17)	471 (±52)	469 (±27)
**6 months**	2.77 (±0.52)	1.93 (±0.20)	718 (±107)	578 (±55)
**9 months**	2.41 (±0.36)	2.11 (±0.26)	832 (±151)	606 (±23)
**12 months**	2.49 (±0.46)	2.12 (±0.10)	708 (±101)	704 (±34)
**CA**	**Protein (% of energy)**		**Fat (% of energy)**	
	Early	Late	Early	Late
	Mean ± SE	Mean ± SE	Mean ± SE	Mean ± SE
**6 weeks**	9.6 (±1.1)	9.4 (±0.7)	45.3 (±1.0)	48.4 (±1.4)
**12 weeks**	8.7 (±0.7)	8.9 (±0.7)	47.1 (±1.3)	48.5 (±1.3)
**6 months**	9.6 (±1.1)	8.5 (±0.4)	40.6 (±1.7)	41.8 (±3.1)
**9 months**	8.9 (±0.1)	10.7 (±1.5)	40.9 (±0.4)	37.9 (±4.0)
**12 months**	10.6 (±0.4)	10.0 (±0.5)	38.5 (±3.3)	37.6 (±3.8)
**CA**	**Carbohydrates (% of energy)**		**Arachidonic Acid (mg/d)**	
	Early	Late	Early	Late
	Mean ± SE	Mean ± SE	Mean ± SE	Mean ± SE
**6 weeks**	45.2 (±0.8)	42.2 (±0.8)	73.0 (±36.5)	110.3 (±9.4)
**12 weeks**	44.2 (±1.0)	42.6 (±0.9)	90.5 (±37.5)	106.4 (±10.2)
**6 months**	49.8 (±1.5)	49.7 (±3.2)	112.7 (±38.3)	79.5 (±18.9)
**9 months**	50.2 (±0.5)	51.4 (±2.8)	134.7 (±33.6)	20.7 (±11.9)
**12 months**	50.9 (±3.4)	52.4 (±3.4)	51.9 (±30.7)	35.9 (±12.2)

CA	Docosahexaenoic Acid (mg/d)		Linoleic Acid (% of energy)	
	Early	Late	Early	Late
	Mean ± SE	Mean ± SE	Mean ± SE	Mean ± SE
**6 weeks**	83.9 (±13.5)	31.2 (±9.6)	6.4 (±1.0)	6.0 (±0.4)
**12 weeks**	83.3 (±28.0)	110.5 (±24.5)	6.6 (±0.7)	5.6 (±0.3)
**6 months**	109.1 (±20.0)	84.9 (±14.4)	6.0 (±0.6)	5.9 (±0.6)
**9 months**	113.3 (±50.7)	23.4 (±12.4)	5.6 (±0.3)	4.7 (±1.5)
**12 months**	56.8 (±38.1)	33.4 (±12.0)	5.8 (±1.0)	4.8 (±0.9)
**CA**	**α-Linolenic Acid (% of energy)**		**LA/ALA ratio (% of energy)**	
	Early	Late	Early	Late
	Mean ± SE	Mean ± SE	Mean ± SE	Mean ± SE
**6 weeks**	0.57 (±0.11)	0.82 (±0.15)	11.25 (±0.72)	8.26 (±1.08)
**12 weeks**	0.69 (±0.12)	0.73 (±0.12)	10.08 (±0.60)	8.45 (±0.94)
**6 months**	1.10 (±0.28)	0.87 (±0.09)	6.12 (±0.94)	7.17 (±1.09)
**9 months**	0.98 (±0.02)	0.81 (±013)	5.66 (±0.41)	5.27 (±1.28)
**12 months**	1.07 (±0.14)	0.79 (±0.11)	5.68 (±0.96)	6.24 (±0.74)

CA: corrected age; SE: standard error. No p-values were calculated between the groups due to the small number of infants with NEC.

2.2.3. Macronutrient intake comparing early and late introduction of solids in infants with IVH

Supplementary Table S8: *Numbers of available dietary records from infants with/without intraventricular hemorrhage ≥ grade II.*

CA	EARLY			LATE		
	total	Infants without BPD, NEC, IVH	IVH	total	Infants without BPD, NEC, IVH	IVH
6 weeks	75	64 (85%)	11 (15%)	42	37 (88%)	5 (12%)
12 weeks	91	79 (87%)	12 (13%)	44	36 (82%)	8 (18%)
6 months	78	66 (85%)	12 (15%)	49	41 (84%)	8 (16%)
9 months	56	48 (86%)	8 (14%)	36	29 (81%)	7 (19%)
12 months	59	48 (86%)	11 (14%)	41	32 (78%)	9 (22%)

CA: corrected age; BPD: bronchopulmonary dysplasia; NEC: necrotizing enterocolitis; IVH: intraventricular hemorrhage. Data are presented as numbers with percentages in parentheses.

Supplementary Table S9: *Nutrient intake comparing early and late introduction of solids in infants with intraventricular hemorrhage ≥ grade II.*

CA	Protein (g/kg/d)			Energy (kcal/d)		
	Early Mean ± SE	Late Mean ± SE	p-adj.	Early Mean ± SE	Late Mean ± SE	p-adj.
6 weeks	2.46 (±0.34)	2.39 (±0.42)	0.97	513 (±33)	414 (±41)	0.50
12 weeks	2.00 (±0.26)	1.71 (±0.16)	0.97	532 (±36)	464 (±27)	0.51
6 months	1.98 (±0.14)	1.89 (±0.17)	0.97	622 (±30)	588 (±44)	0.85
9 months	2.45 (±0.25)	2.02 (±0.08)	0.97	716 (±69)	668 (±31)	0.85
12 months	2.52 (±0.36)	2.34 (±0.17)	0.97	775 (±95)	694 (±32)	0.85

CA	Protein (% of energy)			Fat (% of energy)		
	Early Mean ± SE	Late Mean ± SE	p-adj.	Early Mean ± SE	Late Mean ± SE	p-adj.
6 weeks	8.0 (±0.4)	8.8 (±0.8)	0.96	47.9 (±0.7)	48.3 (±1.7)	0.76
12 weeks	8.1 (±0.4)	7.8 (±0.5)	0.96	47.0 (±1.6)	49.7 (±1.1)	0.68
6 months	8.4 (±0.4)	8.3 (±0.4)	0.96	39.6 (±1.8)	52.6 (±2.3)	0.68
9 months	10.3 (±0.7)	9.8 (±0.5)	0.96	36.5 (±3.1)	39.3 (±2.6)	0.76
12 months	11.0 (±0.8)	11.0 (±0.6)	0.96	34.1 (±1.6)	36.5 (±2.5)	0.68

CA	Carbohydrates (% of energy)			Arachidonic Acid (mg/d)		
	Early Mean ± SE	Late Mean ± SE	p-adj.	Early Mean ± SE	Late Mean ± SE	p-adj.
6 weeks	44.1 (±0.7)	42.9 (±1.1)	0.46	106.8 (±7.5)	87.6 (±12.9)	0.91
12 weeks	44.9 (±1.5)	42.5 (±0.8)	0.46	106.9 (±16.1)	108.9 (±12.6)	0.93
6 months	51.9 (±1.6)	49.1 (±2.3)	0.46	80.1 (±13.1)	88.9 (±12.9)	0.93
9 months	53.2 (±2.5)	50.9 (±2.5)	0.53	87.4 (±19.4)	63.9 (±15.2)	0.91
12 months	54.9 (±1.8)	52.5 (±2.0)	0.46	52.3 (±16.2)	45.4 (±11.0)	0.93

CA	Docosahexaenoic Acid (mg/d)			Linoleic Acid (% of energy)		
	Early Mean ± SE	Late Mean ± SE	p-adj.	Early Mean ± SE	Late Mean ± SE	p-adj.
**6 weeks**	103.0 (±8.7)	70.1 (±12.1)	0.25	6.42 (±0.20)	5.68 (±0.44)	0.48
**12 weeks**	100.4 (±15.6)	84.1 (±9.1)	0.47	6.41 (±0.33)	5.45 (±0.18)	0.48
**6 months**	72.0 (±11.4)	91.3 (±10.5)	0.42	5.51 (±0.49)	5.59 (±0.41)	0.98
**9 months**	107.9 (±33.3)	72.9 (±24.1)	0.42	4.88 (±0.47)	4.90 (±0.69)	0.98
**12 months**	57.1 (±12.7)	44.5 (±13.9)	0.47	4.89 (±0.50)	4.50 (±063)	0.98

CA	α-Linolenic Acid (% of energy)			LA/ALA ratio (% of energy)		
	Early Mean ± SE	Late Mean ± SE	p-adj.	Early Mean ± SE	Late Mean ± SE	p-adj.
**6 weeks**	0.80 (±0.08)	0.76 (±0.17)	0.97	8.64 (±077)	8.62 (±1.3)	0.73
**12 weeks**	0.83 (±0.8)	0.64 (±0.08)	0.66	8.28 (±0.79)	9.21 (±0.78)	0.73
**6 months**	0.85 (±0.10)	0.80 (±0.08)	0.97	6.95 (±0.60)	7.52 (±0.85)	0.73
**9 months**	1.21 (±.0.59)	0.79 (±0.10)	0.97	6.81 (±1.18)	6.50 (±0.72)	0.73
**12 months**	0.72 (±0.05)	0.72 (±0.09)	0.97	6.92 (±0.59)	6.59 (±0.71)	0.73

CA: corrected age; SE: standard error. p-adj. <0.05 were considered statistically significant and marked bold.

3. Subgroup analysis: macronutrient intake in breastfed, formula-fed, mixed-fed infants and infants without milk-feeding

Nutrient intake was assessed by comparing breastfed with formula-fed, mixed-fed and infants who received neither breastmilk nor formula (=no milk). To detect differences between the type of feeding, students t-test or Mann-Whitney-U-test were applied (breastfed vs. formula, mixed, none). Statistical testing was not performed for mixed-fed infants at 9 and 12 months CA and “no milk” from 6 weeks – 6 months CA, due to the small sample size. Standard errors and adjusted p-values were calculated to test the null hypothesis of no difference between the type of feeding. Statistical significance was set at p-adj. < 0.05. As an additional analysis, the p-values for between-type of feeding comparisons of the same nutrient at different timepoints were adjusted using the Bonferroni-Holm method. The statistical analysis was performed using R version 4.1.1 (R Core Team, 2022).

Supplementary Table S10*: Nutrient intake comparing breastfed, formula-fed, mixed-fed and no milk intake.*

CA	Protein (g/kg/d)						
	**Breastfed Mean ± SE**	**Formula Mean ± SE**	**p-adj.**	**Mixed Mean ± SE**	**p-adj.**	**No milk Mean ± SE**	**p-adj.**
**6 weeks**	2.24 (±0.09)	2.44 (±0.09)	0.90	2.61 (±0.17)	0.16		
**12 weeks**	1.72 (±0.05)	1.99 (±0.06)	0.07	1.93 (±0.06)	**0.03**	/	
**6 months**	1.87 (±0.07)	2.18 (±0.07)	0.07	2.11 (±0.21)	0.99		
**9 months**	1.99 (±0.10)	2.47 (±0.07)	**0.02**	3.15 (±0.56)	/	3.05 (±0.56)	**0.03**
**12 months**	2.48 (±0.14)	2.67 (±0.10)	0.99	2.24 (±0.33)	/	2.29 (±0.11)	0.26

Recommended daily protein intake: 1.6 g/kg/d.

CA	Energy (kcal/d)						
	**Breastfed Mean ± SE**	**Formula Mean ± SE**	**p-adj.**	**Mixed Mean ± SE**	**p-adj.**	**No milk Mean ± SE**	**p-adj.**
**6 weeks**	506 (±14)	504 (±11)	0.70	488 (±19)	0.42		
**12 weeks**	535 (±9)	534 (±11)	0.70	532 (±12)	0.70	/	
**6 months**	649 (±15)	631 (±15)	0.41	643 (±25)	0.70		
**9 months**	690 (±27)	748 (±15)	0.32	967 (±144)	/	732 (±61)	0.70
**12 months**	847 (±38)	814 (±21)	0.70	846 (±96)	/	743 (±32)	0.32

Recommended daily energy intake: 0-3 months: 500-550 kcal/d; 4-12 months: 600-700 kcal/d.

CA	Protein (% energy)						
	**Breastfed Mean ± SE**	**Formula Mean ± SE**	**p-adj.**	**Mixed Mean ± SE**	**p-adj.**	**No milk Mean ± SE**	**p-adj.**
**6 weeks**	7.4 (±0.2)	8.6 (±0.2)	**<0.001**	8.4 (±0.2)	**0.003**		
**12 weeks**	6.8 (±0.1)	8.3 (±0.1)	**<0.001**	7.6 (±0.2)	**<0.001**	/	
**6 months**	7.8 (±0.2)	9.5 (±0.2)	**<0.001**	9.2 (±0.7)	**0.04**		
**9 months**	9.6 (±0.3)	10.6 (±0.2)	**0.04**	9.4 (±1.0)	/	13.1 (±1.0)	**0.002**
**12 months**	10.9 (±0.4)	11.4 (±0.3)	0.68	9.6 (±1.0)	/	14.0 (±0.6)	**0.003**

Recommended daily protein intake: <15% of energy.

CA	Fat (% energy)						
	**Breastfed Mean ± SE**	**Formula Mean ± SE**	**p-adj.**	**Mixed Mean ± SE**	**p-adj.**	**No milk Mean ± SE**	**p-adj.**
**6 weeks**	50.8 (±0.6)	47.3 (±0.2)	**<0.001**	48.3 (±0.6)	**<0.001**		
**12 weeks**	52.1 (±0.1)	46.3 (±0.3)	**<0.001**	49.2 (±0.4)	**<0.001**	/	
**6 months**	46.0 (±0.8)	39.4 (±0.6)	**<0.001**	42.8 (±1.3)	0.05		
**9 months**	38.0 (±1.3)	36.2 (±0.7)	0.30	37.1 (±2.0)	/	33.9 (±3.6)	0.28
**12 months**	34.8 (±1.4)	35.4 (±0.5)	0.63	40.4 (±4.4)	/	34.8 (±2.0)	0.58

Recommended daily fat intake 0-3 months: 45-50% of energy; 4-12 months: 35-45% of energy

CA	Carbohydrates (% of energy)						
	**Breastfed Mean ± SE**	**Formula Mean ± SE**	**p-adj.**	**Mixed Mean ± SE**	**p-adj.**	**No milk Mean ± SE**	**p-adj.**
**6 weeks**	41.8 (±0.3)	44.1 (±0.2)	**<0.001**	43.3 (±0.5)	**0.002**		
**12 weeks**	41.1 (±0.2)	45.4 (±0.3)	**<0.001**	43.2 (±0.4)	**<0.001**	/	
**6 months**	46.2 (±0.7)	51.1 (±0.5)	**<0.001**	48.0 (±0.9)	0.86		
**9 months**	52.3 (±2.0)	53.3 (±0.7)	0.55	53.4 (±2.4)	/	53.0 (±2.9)	1.00
**12 months**	54.3 (±1.2)	53.2 (±0.5)	0.53	49.9 (±3.4)	/	53.1 (±1.3)	0.52

/

CA	Arachidonic Acid (mg/d)

	Breastfed Mean ± SE	Formula Mean ± SE	p-adj.	Mixed Mean ± SE	p-adj.	No milk Mean ± SE	p-adj.
**6 weeks**	133 (±4)	108 (±5)	**0.001**	102 (±7)	**<0.001**		
**12 weeks**	143 (±3)	109 (±4)	**<0.001**	119 (±7)	**<0.001**	/	
**6 months**	131 (±4)	86 (±4)	**<0.001**	117 (±9)	0.27		
**9 months**	97 (±7)	82 (±5)	**0.02**	81 (±11)	/	35 (±17)	**0.008**
**12 months**	81 (±8)	79 (±7)	0.37	108 (±29)	/	41 (±9)	**0.001**

Recommended daily AA intake: 0-6 months: 140 mg/d; 6-12 months: no recommendations exist.

CA	Docosahexaenoic Acid (mg/d)						
	Breastfed Mean ± SE	Formula Mean ± SE	p-adj.	Mixed Mean ± SE	p-adj.	No milk Mean ± SE	p-adj.
**6 weeks**	90 (±3)	107 (±3)	0.06	86 (±6)	0.48		
**12 weeks**	98 (±2)	112 (±4)	0.15	103 (±6)	0.49	/	
**6 months**	101 (±6)	99 (±5)	0.48	100 (±8)	0.72		
**9 months**	78 (±9)	96 (±8)	0.48	79 (±16)	/	41 (±18)	0.26
**12 months**	64 (±8)	84 (±6)	0.48	86 (±28)	/	84 (±17)	0.06

Recommended daily DHA intake: 0-12 months: 100 mg/d.

CA	Linoleic Acid (% of energy)						
	Breastfed Mean ± SE	Formula Mean ± SE	p-adj.	Mixed Mean ± SE	p-adj.	No milk Mean ± SE	p-adj.
**6 weeks**	5.21 (±0.06)	6.58 (±0.13)	**<0.001**	6.20 (±0.20)	**<0.001**		
**12 weeks**	5.29 (±0.04)	6.57 (±0.09)	**<0.001**	5.97 (±0.13)	**<0.001**	/	
**6 months**	5.22 (±0.11)	5.77 (±0.11)	**0.01**	4.50 (±0.21)	0.54		
**9 months**	4.74 (±0.13)	5.13 (±0.11)	0.16	5.87 (±0.25)	/	3.69 (±0.64)	0.16
**12 months**	4.48 (±0.30)	5.01 (±0.11)	0.29	4.87 (±0.65)	/	4.27 (±0.29)	0.67

Recommended daily LA intake: 0-3 months: 4.0% of energy; 4-12 months: 3.5% of energy.

CA	α-Linolenic Acid (% of energy)						
	Breastfed Mean ± SE	Formula Mean ± SE	p-adj.	Mixed Mean ± SE	p-adj.	No milk Mean ± SE	p-adj.
**6 weeks**	0.49 (±0.02)	0.82 (±0.03)	**<0.001**	0.74 (±0.05)	**<0.001**		
**12 weeks**	0.48 (±0.01)	0.83 (±0.02)	**<0.001**	0.69 (±0.02)	**<0.001**	/	
**6 months**	0.70 (±0.09)	0.89 (±0.03)	**<0.001**	0.78 (±0.07)	**0.007**		
**9 months**	0.70 (±0.04)	0.88 (±0.06)	**0.002**	0.59 (±0.10)	/	0.64 (±0.10)	0.78
**12 months**	0.70 (±0.09)	0.77 (±0.03)	0.15	0.67 (±0.20)	/	0.71 (±0.08)	0.25

Recommended daily ALA intake: 0-12 months: 0.5 % of energy

CA	LA/ALA ratio						
	Breastfed Mean ± SE	Formula Mean ± SE	p-adj.	Mixed Mean ± SE	p-adj.	No milk Mean ± SE	p-adj.
**6 weeks**	10.8 (±0.06)	6.58 (±0.12)	**<0.001**	6.20 (±0.20)	**<0.001**		
**12 weeks**	5.29 (±0.04)	6.57 (±0.09)	**<0.001**	5.97 (±0.13)	**<0.001**	/	
**6 months**	5.22 (±0.11)	5.77 (±0.11)	**<0.001**	5.50 (±0.22)	0.01		
**9 months**	4.74 (±0.13)	5.13 (±0.11)	**0.02**	5.87 (±0.25)	/	3.69 (±0.64)	0.13
**12 months**	4.47 (±0.30)	5.01 (±0.12)	0.74	4.87 (±0.65)	/	4.31 (±0.31)	0.74

Recommended daily LA/ALA ratio: 0-3 months: 8:1; 4-12 months: 7:1.

CA: corrected age; AA: arachidonic acid;. DHA: docosahexaenoic acid; LA: linoleic acid; ALA: α-linolenic acid; SE: standard error; p-adj. <0.05 were considered statistically significant and marked bold. No milk: infants that received neither human milk nor infant formula.

3.1. Macronutrient intake comparing early and late introduction of solid foods in breastfed, formula-fed, mixed-fed infants and infants without milk-feeding

Nutrient intake was evaluated by comparing the timepoint of solid food introduction (early vs. late) in breastfed, formula-fed, mixed-fed and infants who received neither breastmilk nor formula (=no milk). To detect differences between study subgroups, students t-test or Mann-Whitney-U-test were applied. Statistical testing was not performed for breastfed infants at 12 months CA, mixed-fed infants at 9 and 12 months CA and "no milk fed" at 6 weeks - 9 months CA, due to small sample size. As an additional analysis, the p-values for between-group comparisons of the same nutrient at different timepoints were adjusted using the Bonferroni-Holm method. Standard errors and adjusted p-values were calculated to test the null hypothesis of no difference between the groups. Statistical significance was set at p-adj. < 0.05. The statistical analysis was performed using R Studio (Core Team, 2022).

***Supplementary Table S11**: Numbers of protocols according to type of feeding in the early and late group.*

CA	EARLY					LATE				
	total	breastfed	formula	mixed	no milk	total	breastfed	formula	mixed	no milk
**6 weeks**	86	15 (17%)	62 (72%)	9 (11%)	0 (%)	60	23 (38%)	27 (45%)	10 (17%)	0 (%)
**12 weeks**	106	18 (17%)	75 (71%)	13 (12%)	0 (%)	64	18 (28%)	31 (49%)	15 (23%)	0 (%)
**6 months**	90	17 (19%)	66 (73%)	7 (8%)	0 (%)	66	18 (27%)	37 (56%)	11 (17%)	0 (%)
**9 months**	63	11 (17%)	48 (76%)	1 (2%)	3 (5%)	51	11 (21%)	35 (69%)	2 (4%)	3 (6%)
**12 months**	65	3 (5%)	49 (75%)	1 (2%)	12 (18%)	57	8 (14%)	35 (61%)	2 (4%)	12 (21%)

Data are presented as numbers with percentages in parentheses. Mixed: breastmilk + formula. No milk: infants that received neither human milk nor infant formula.

***Supplementary Table S12:** Overview of different formulas used in this observational study.*

Type of formula	6 weeks CA		12 weeks CA		6 months CA		9 months CA		12 months CA	
	formula (n=87)	mixed (n=21)	formula (n=106)	mixed (n=28)	formula (n=103)	mixed (n=18)	formula (n=83)	mixed (n=3)	formula (n=84)	mixed (n=3)
**Preterm Formula**	19 (21.8%)	6 (28.5%)	7 (6.6%)	3 (10.7%)	1 (1%)	1 (5.6%)	0	0	0	0
**Infant Formula**	64 (73.6%)	11 (52.4%)	89 (83.9%)	23 (82.2%)	84 (81.6%)	16 (88.8%)	61 (73.5%)	3 (100%)	47 (55.9%)	0
**Follow-on Formula**	1 (1.1%)	0	3 (2.8%)	0	8 (7.6%)	1 (5.6%)	18 (21.7%)	0	30 (35.7%)	3 (100%)
**More than one type of formula**	1 (1.1%)*	0	2 (1.9%)*	0	4 (3.9%)	0	1 (1.2%)°	0	0	0
**Other FSPM***	2 (2.3%)	4 (19.0%)	5 (4.7%)	2 (7.1%)	6 (5.8%)	0	2 (2.4%)	0	2 (2.4%)	0
**Toddler Milk 1+**	0	0	0	0	0	0	1 (1.2%)	0	5 (6.0%)	0

*FSMP: Food for special medical purpose; including antireflux formula, high-protein formula, energy-dense formula, formula for infants with cows-milk allergy
CA: corrected age

Supplementary Table S13: *Breastfed infants that received fortification.*

	6 weeks CA		12 weeks CA		6 months CA		9 months CA		12 months CA	
	breastfed (n=38)	mixed (n=21)	breastfed (n=36)	mixed (n=28)	breastfed (n=35)	mixed (n=18)	breastfed (n=22)	mixed (n=3)	breastfed (n=11)	mixed (n=3)
**Mothers Own Milk + Fortification**	7 (18%)	1 (4.8%)	2 (6%)	0	0	0	0	0	0	0

CA: corrected age.

Supplementary Table S14: *Range of nutrients of infant formulas that were additionally entered into the nutritional software due to missing data or changes in formulation over time.*

Nutrient	Unit	Preterm Formula	Infant Formula		Follow-on Formula		Antireflux Formula
			Pre Stage	Stage 1	Stage 2	Stage 3	
**Energy**	kcal	73-75	65-67	66-69	67-70	66-70	66-67
**Protein**	g	2	1.2-1.6	1.2-1.7	1.1-1.7	1.3-1.5	1.3-1.6
**Carbohydrates**	g	7.5-7.7	6.9-7.8	7.2-8.1	7.7-8.5	7.2-8.5	6.8-7.8
**Fat**	g	3.8-4.0	3.3-3.6	3.4-3.6	3.0-3.5	2.9-3.7	3.4-3.5
**Linoleic Acid**	g	0.46-0.7	0.4-0.6	0.4-0.6	0.39-0.70	0.39-0.7	0.5-0.7
**Linolenic Acid**	g	0.06-1	0.05-0.08	0.04-0.09	0.05-0.08	0.05-0.08	0.065-0.09
**Docosahexaenoic Acid**	mg	14.4-18.1	6.4-17	7-17	0-17	4-17	0-8
**Arachidonic Acid**	mg	14.4-20	6.5-17	7-17	0-9	0-9	0-12

Supplementary Table S15: *Nutrient intake comparing early and late introduction of solid foods in breastfed, formula-fed, mixed-fed infants and without milk-feeding.*

Protein (g/kg/d)

CA	Breastfed			Formula			Mixed			No milk		
	Early	Late	p-adj.	Early	Late	p-adj.	Early	Late	p-adj.	Early	Late	p-adj.
	Mean ± SE	Mean ± SE		Mean ± SE	Mean ± SE		Mean ± SE	Mean ± SE		Mean ± SE	Mean ± SE	
**6 weeks**	2.4 (±0.2)	2.1 (±0.1)	0.49	2.5 (±0.1)	2.3 (±0.2)	0.36	2.4 (±0.3)	2.8 (±0.2)	0.49			
**12 weeks**	1.8 (±0.1)	1.6 (±0.1)	0.36	2.0 (±0.1)	1.9 (±0.1)	0.49	1.9 (±0.1)	2.0 (±0.1)	0.49	/		
**6 months**	1.9 (±0.1)	1.8 (±0.1)	0.68	2.3 (±0.1)	2.0 (±0.1)	0.36	2.7 (±0.4)	1.8 (±0.2)	0.36			
**9 months**	1.9 (±0.1)	2.1 (±0.2)	0.52	2.4 (±0.1)	2.5 (±0.1)	0.90	3.7 (±/)	3.1 (±1.4)	/	3.8 (±1.0)	2.4 (±0.2)	/
**12 months**	1.9 (±0.2)	2.7 (±0.1)	/	2.7 (±0.1)	2.6 (±0.2)	0.68	1.9 (±/)	2.4 (±0.5)	/	3.1 (±0.2)	2.8 (±0.3)	0.65

Energy (kcal/d)

CA	Breastfed			Formula			Mixed			No milk		
	Early	Late	p-adj.	Early	Late	p-adj.	Early	Late	p-adj.	Early	Late	p-adj.
	Mean ± SE	Mean ± SE		Mean ± SE	Mean ± SE		Mean ± SE	Mean ± SE		Mean ± SE	Mean ± SE	
**6 weeks**	534 (±23)	488 (±17)	0.11	519 (±14)	473 (±19)	0.11	474 (±25)	500 (±31)	0.56			
**12 weeks**	545 (±9)	525 (±14)	0.22	550 (±13)	496 (±23)	0.11	532 (±18)	533 (±17)	0.66	/		
**6 months**	678 (±20)	621 (±22)	0.43	660 (±20)	582 (±18)	0.11	722 (±42)	592 (±22)	0.11			
**9 months**	664 (±42)	717 (±32)	0.11	768 (±21)	723 (±21)	0.23	855 (±/)	1023 (±229)	/	838 (±88)	627 (±7)	/
**12 months**	725 (±66)	893 (±37)	/	822 (±28)	801 (±30)	0.11	838 (±/)	851 (±166)	/	719 (±49)	766 (±42)	0.55

Protein (% of energy)

CA	Breastfed			Formula			Mixed			No milk		
	Early	Late	p-adj.	Early	Late	p-adj.	Early	Late	p-adj.	Early	Late	p-adj.
	Mean ± SE	Mean ± SE		Mean ± SE	Mean ± SE		Mean ± SE	Mean ± SE		Mean ± SE	Mean ± SE	
**6 weeks**	7.5 (±0.4)	7.4 (±0.3)	0.59	8.7 (±0.2)	8.5 (±0.4)	0.49	8.3 (±0.3)	8.5 (±0.4)	0.49			
**12 weeks**	6.8 (±0.2)	6.8 (±0.2)	0.78	8.3 (±0.1)	8.3 (±0.2)	0.59	7.4 (±0.2)	7.8 (±0.3)	0.49	/		
**6 months**	8.1 (±0.3)	7.5 (±0.3)	0.44	9.6 (±0.2)	9.4 (±0.4)	0.44	10.8 (±1.5)	8.2 (±0.3)	0.44			
**9 months**	9.9 (±0.4)	9.3 (±0.5)	0.52	10.5 (±0.2)	10.6 (±0.4)	0.59	10.6 (±/)	8.9 (±1.5)	/	14.2 (±1.4)	12.1 (±1.4)	/
**12 months**	11.0 (±0.4)	10.9 (±0.5)	/	11.7 (±0.4)	11.0 (±0.3)	0.44	8.0 (±/)	10.4 (±0.9)	/	15.3 (±0.8)	12.7 (±0.8)	0.26

Fat (% of energy)

CA	Breastfed			Formula			Mixed			No milk		
	Early	Late	p-adj.	Early	Late	p-adj.	Early	Late	p-adj.	Early	Late	p-adj.
	Mean ± SE	Mean ± SE		Mean ± SE	Mean ± SE		Mean ± SE	Mean ± SE		Mean ± SE	Mean ± SE	
**6 weeks**	50.5 (±0.9)	51.0 (±0.8)	0.86	47.3 (±0.2)	47.2 (±0.4)	0.66	47.3 (±1.1)	49.2 (±0.5)	0.31			
**12 weeks**	51.8 (±0.5)	52.4 (±0.5)	0.13	45.9 (±0.4)	47.2 (±0.3)	0.08	48.4 (±0.8)	49.9 (±0.4)	0.29	/		
**6 months**	44.7 (±1.3)	47.3 (±1.0)	0.30	38.0 (±0.7)	41.7 (±1.0)	**0.01**	39.0 (±2.2)	45.1 (±1.1)	0.08			
**9 months**	38.6 (±2.0)	37.5 (±1.8)	0.73	35.6 (±0.9)	36.9 (±1.0)	0.45	34.4 (±/)	38.5 (±2.4)	/	29.6 (±2.4)	38.2 (±6.3)	/
**12 months**	37.0 (±3.4)	33.9 (±1.5)	/	35.2 (±0.8)	35.8 (±0.64)	0.70	48.0 (±/)	36.6 (±3.8)	/	33.8 (±1.4)	31.9 (±2.0)	0.44

Carbohydrates (% of energy)

CA	Breastfed			Formula			Mixed			No milk		
	Early	Late	p-adj.	Early	Late	p-adj.	Early	Late	p-adj.	Early	Late	p-adj.
	Mean ± SE	Mean ± SE		Mean ± SE	Mean ± SE		Mean ± SE	Mean ± SE		Mean ± SE	Mean ± SE	
**6 weeks**	42.0 (±0.5)	41.6 (±0.4)	0.52	44.0 (±0.2)	44.3 (±0.4)	0.43	44.4 (±0.8)	42.4 (±0.4)	0.26			
**12 weeks**	41.3 (±0.3)	40.9 (±0.3)	0.39	45.8 (±0.4)	44.5 (±0.2)	0.35	44.2 (±0.7)	42.3 (±0.3)	0.20	/		
**6 months**	47.2 (±1.2)	45.2 (±0.9)	0.35	52.3 (±0.6)	49.0 (±0.9)	**0.01**	50.2 (±1.4)	46.6 (±1.0)	0.22			
**9 months**	51.5 (±1.7)	53.2 (±1.8)	0.53	54.0 (±0.9)	52.5 (±1.0)	0.39	55.0 (±/)	52.6 (±3.9)	/	56.2 (±2.7)	49.7 (±4.9)	/

**12 months**	52.0 (±3.0)	55.2 (±1.2)	/	53.1 (±0.9)	53.3 (±0.6)	0.88	44.0 (±/)	52.9 (±2.9)	/	50.9 (±1.7)	55.4 (±1.9)	0.26

CA	**Arachidonic Acid (mg/d)**											
	**Breastfed**			**Formula**			**Mixed**			**No milk**		
	**Early**	**Late**	**p-adj.**	**Early**	**Late**	**p-adj.**	**Early**	**Late**	**p-adj.**	**Early**	**Late**	**p-adj.**
	**Mean ± SE**	**Mean ± SE**		**Mean ± SE**	**Mean ± SE**		**Mean ± SE**	**Mean ± SE**		**Mean ± SE**	**Mean ± SE**	
**6 weeks**	140 (±7)	130 (±5)	0.85	109 (±5)	104 (±9)	0.85	97 (±10)	106 (±10)	0.85			
**12 weeks**	145 (±3)	141 (±5)	0.85	106 (±6)	117 (±7)	0.85	116 (±9)	121 (±10)	0.85	/		
**6 months**	127 (±6)	136 (±6)	0.85	81 (±5)	95 (±7)	0.85	117 (±9)	117 (±20)	0.98			
**9 months**	91.9 (±11)	102 (±8)	0.85	91 (±9)	69 (±7)	0.85	59 (±/)	92 (±4)	/	21 (±10)	49 (±35)	/
**12 months**	78 (±16)	82 (±10)	/	83 (±11)	75 (±7)	0.85	163 (±/)	80 (±13)	/	44 (±8)	39 (±10)	0.85

CA	**Docosahexaenoic Acid (mg/d)**											
	**Breastfed**			**Formula**			**Mixed**			**No milk**		
	**Early**	**Late**	**p-adj.**	**Early**	**Late**	**p-adj.**	**Early**	**Late**	**p-adj.**	**Early**	**Late**	**p-adj.**
	**Mean ± SE**	**Mean ± SE**		**Mean ± SE**	**Mean ± SE**		**Mean ± SE**	**Mean ± SE**		**Mean ± SE**	**Mean ± SE**	
**6 weeks**	95 (±7)	89 (±4)	0.76	108 (±5)	104 (±7)	0.76	79 (±8.3)	91.6 (±7.8)	0.76			
**12 weeks**	99 (±2)	97 (±3)	0.76	111 (±5)	114 (±7)	0.85	94 (±18)	110 (±10)	0.76	/		
**6 months**	101 (±6)	101 (±9)	0.76	97 (±6)	102 (±8)	0.76	108 (±16)	96 (±8)	0.76			
**9 months**	86 (±18)	71 (±5)	0.86	99 (±12)	91 (±8)	0.76	46 (±/)	95 (±1)	/	44 (±33)	39 (±24)	/
**12 months**	72 (±19)	60 (±10)	/	79 (±7)	90 (±9)	0.76	140 (±/)	59 (±11)	/	70 (±27)	30 (±8)	0.76

CA	**Linoleic Acid (% of energy)**											
	**Breastfed**			**Formula**			**Mixed**			**No milk**		
	**Early**	**Late**	**p-adj.**	**Early**	**Late**	**p-adj.**	**Early**	**Late**	**p-adj.**	**Early**	**Late**	**p-adj.**
	**Mean ± SE**	**Mean ± SE**		**Mean ± SE**	**Mean ± SE**		**Mean ± SE**	**Mean ± SE**		**Mean ± SE**	**Mean ± SE**	
**6 weeks**	5.18 (±0.11)	5.18 (±0.08)	0.96	6.58 (±0.11)	6.59 (±0.16)	0.96	6.05 (±0.32)	6.33 (±0.26)	0.96			
**12 weeks**	5.28 (±0.05)	5.29 (±0.05)	0.96	6.60 (±0.11)	6.48 (±0.11)	0.96	5.95 (±0.14)	5.99 (±0.22)	0.96	/		
**6 months**	5.31 (±0.21)	5.13 (±0.12)	0.96	5.70 (±0.14)	5.89 (±0.17)	0.96	5.48 (±0.50)	5.51 (±0.20)	0.96			
**9 months**	4.82 (±0.30)	4.66 (±0.29)	0.96	5.16 (±0.15)	5.09 (±0.18)	0.96	5.39 (±/)	6.11 (±0.15)	/	4.02 (±0.52)	3.37 (±1.30)	/
**12 months**	4.93 (±0.15)	4.31 (±0.40)	/	4.95 (±0.16)	5.09 (±0.16)	0.96	6.08 (±/)	4.26 (±0.38)	/	4.66 (±0.45)	3.88 (±0.37)	0.96

CA	**α-Linolenic Acid (% of energy)**											
	**Breastfed**			**Formula**			**Mixed**			**No milk**		
	**Early**	**Late**	**p-adj.**	**Early**	**Late**	**p-adj.**	**Early**	**Late**	**p-adj.**	**Early**	**Late**	**p-adj.**
	**Mean ± SE**	**Mean ± SE**		**Mean ± SE**	**Mean ± SE**		**Mean ± SE**	**Mean ± SE**		**Mean ± SE**	**Mean ± SE**	
**6 weeks**	0.53 (±0.05)	0.07 (±0.01)	0.61	0.85 (±0.02)	0.75 (±0.03)	0.13	0.66 (±0.05)	0.80 (±0.07)	0.81			
**12 weeks**	0.49 (±0.02)	0.47 (±0.00)	0.68	0.84 (±0.02)	0.81 (±0.03)	0.70	0.72 (±0.04)	0.66 (±0.03)	0.70	/		
**6 months**	0.68 (±0.07)	0.73 (±0.15)	0.61	0.91 (±0.04)	0.84 (±0.02)	0.92	0.88 (±0.18)	0.72 (±0.05)	0.89			
**9 months**	0.76 (±0.17)	0.64 (±0.06)	0.92	0.93 (±1.0)	0.80 (±0.05)	0.70	0.42 (±/)	0.68 (±0.11)	/	0.52 (±0.16)	0.75 (±0.15)	/
**12 months**	0.96 (±0.28)	0.59 (±0.05)	/	0.79 (±0.03)	0.74 (±0.04)	0.70	1.08 (±/)	0.47 (±0.03)	/	0.66 (±0.12)	0.62 (±0.06)	0.89

CA	**LA/ALA ratio (% of energy)**											
	**Breastfed**			**Formula**			**Mixed**			**No milk**		
	**Early**	**Late**	**p-adj.**	**Early**	**Late**	**p-adj.**	**Early**	**Late**	**p-adj.**	**Early**	**Late**	**p-adj.**
	**Mean ± SE**	**Mean ± SE**		**Mean ± SE**	**Mean ± SE**		**Mean ± SE**	**Mean ± SE**		**Mean ± SE**	**Mean ± SE**	
**6 weeks**	10.4 (0.5)	11.1 (±0.1)	0.33	8.1 (±0.2)	9.1 (±0.4)	0.20	9.3 (±0.4)	8.4 (±0.6)	0.33			
**12 weeks**	10.7 (±0.3)	11.2 (±0.0)	0.09	7.9 (±0.2)	8.3 (±0.3)	0.39	8.5 (±0.4)	9.1 (±0.3)	0.33	/		
**6 months**	8.5 (±0.6)	9.1 (±0.6)	0.33	6.7 (±0.2)	7.1 (±0.3)	0.36	6.9 (±0.7)	7.9 (±0.4)	0.33			
**9 months**	7.8 (±0.8)	7.7 (±0.6)	0.96	6.30 (±0.3)	6.7 (±0.3)	0.43	12.7 (±/)	9.2 (±1.7)	/	9.5 (±3.4)	4.2 (±1.1)	/
**12 months**	6.0 (±1.3)	7.3 (±0.4)	/	6.7 (±0.2)	7.3 (±0.3)	0.33	5.7 (±/)	9.2 (±0.1)	/	8.3 (±1.3)	6.9 (±0.8)	0.56

CA: corrected age; SE: standard error. p-adj. <0.05 were considered statistically significant and marked bold. No milk: infants that received neither human milk nor infant formula.
